# The role of alpha-synuclein in synucleinopathy: Impact on lipid regulation at mitochondria–ER membranes

**DOI:** 10.1038/s41531-025-00960-x

**Published:** 2025-04-30

**Authors:** Peter A. Barbuti, Cristina Guardia-Laguarta, Taekyung Yun, Zena K. Chatila, Xena Flowers, Chantel Wong, Bruno F. R. Santos, Simone B. Larsen, James S. Lotti, Nobutaka Hattori, Elizabeth Bradshaw, Ulf Dettmer, Saranna Fanning, Vilas Menon, Hasini Reddy, Andrew F. Teich, Rejko Krüger, Estela Area-Gomez, Serge Przedborski

**Affiliations:** 1 Department of Neurology, Columbia University Irving Medical Center, New York, NY USA; 2 Center for Motor Neuron Biology and Diseases, Columbia University Irving Medical Center, New York, NY USA; 3 Translational Neuroscience, Luxembourg Centre for Systems Biomedicine, University of Luxembourg, Belval, Luxembourg; 4 Transversal Translational Medicine, Luxembourg Institute of Health, Luxembourg City, Luxembourg; 5 Center for Biological Research (CIB), - Margarita Salas, CSIC, Madrid, Spain; 6 Taub Institute for Research on Alzheimer’s Disease and the Aging Brain, Columbia University, New York, NY USA; 7 The Carol and Gene Ludwig Center for Research on Neurodegeneration, Columbia University, New York, NY USA; 8 Department of Neuroscience, Barnard College of Columbia University, New York, NY USA; 9 Disease Modelling and Screening Platform, Luxembourg Centre for Systems Biomedicine, University of Luxembourg, Belval, Luxembourg; 10 Ann Romney Center for Neurologic Diseases, Brigham and Women’s Hospital and Harvard Medical School, Boston, MA USA; 11 Department of Neurology, Juntendo University School of Medicine, Bunkyo-ku, Tokyo Japan; 12 Center for Translational and Computational Neuroimmunology, Columbia University, New York, NY USA; 13 Department of Pathology & Cell Biology, Columbia University Irving Medical Center, New York, NY USA; 14 Department of Neuroscience, Columbia University, New York, NY USA

**Keywords:** Cellular neuroscience, Parkinson's disease

## Abstract

The protein alpha-synuclein (αSyn) plays a pivotal role in the pathogenesis of synucleinopathies, including Parkinson’s disease and multiple system atrophy, with growing evidence indicating that lipid dyshomeostasis is a key phenotype in these neurodegenerative disorders. Previously, we identified that αSyn localizes, at least in part, to mitochondria-associated endoplasmic reticulum membranes (MAMs), which are transient functional domains containing proteins that regulate lipid metabolism, including the de novo synthesis of phosphatidylserine. In the present study, we analyzed the lipid composition of postmortem human samples, focusing on the substantia nigra pars compacta of Parkinson’s disease and controls, as well as three less affected brain regions of Parkinson’s donors. To further assess synucleinopathy-related lipidome alterations, similar analyses were performed on the striatum of multiple system atrophy cases. Our data reveal region- and disease-specific changes in the levels of lipid species. Specifically, our data revealed alterations in the levels of specific phosphatidylserine species in brain areas most affected in Parkinson’s disease. Some of these alterations, albeit to a lesser degree, are also observed in multiple system atrophy. Using induced pluripotent stem cell-derived neurons, we show that αSyn regulates phosphatidylserine metabolism at MAM domains, and that αSyn dosage parallels the perturbation in phosphatidylserine levels. These findings support the notion that αSyn pathophysiology is linked to the dysregulation of lipid homeostasis, which may contribute to the vulnerability of specific brain regions in synucleinopathy. These findings have significant therapeutic implications.

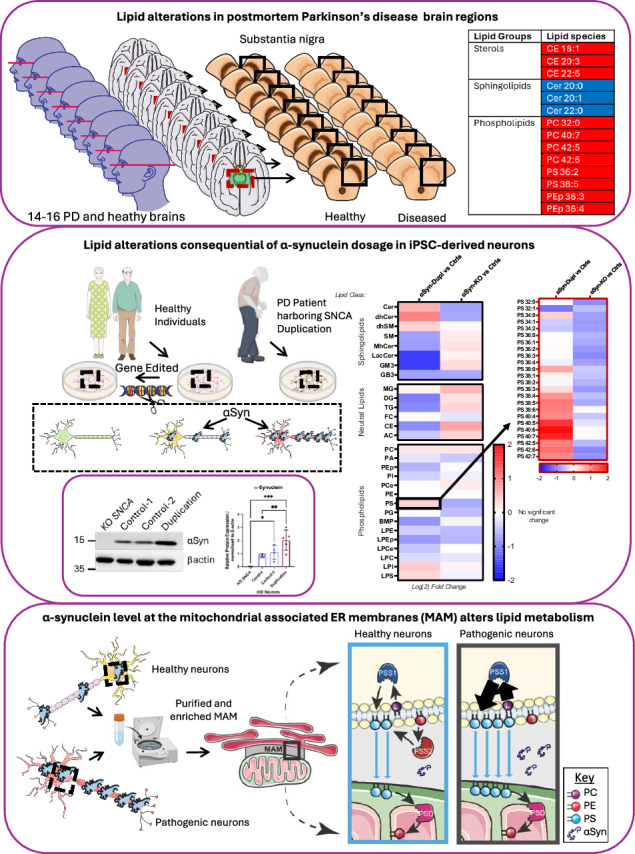

## Introduction

Synucleinopathy refers to a cluster of adult-onset neurodegenerative conditions marked by a buildup of alpha-synuclein (αSyn) protein aggregates within neuronal cell bodies and fibers, and occasionally within glial cells^1^. These disorders primarily encompass Parkinson’s disease (PD), dementia with Lewy bodies, and multiple system atrophy (MSA)^1^. They manifest in various motor and cognitive impairments for which there is currently no cure and a lack of effective disease-modifying treatments. Besides αSyn being used as a biomarker for diagnosis of synucleinopathy^2,3^, a host of genetic studies have provided evidence of its role in synucleinopathy-related neurodegeneration^4^. Polymorphisms in the gene encoding αSyn, *SNCA*, that result in increased αSyn expression, have been associated with sporadic PD development^5,6^, and genomic *SNCA* multiplication and rare dominantly inherited *SNCA* point mutations (e.g., A30P and A53T) with familial PD^4^. The duplication or triplication of the *SNCA* locus results in αSyn expression at 1.5× or 2× the level observed for a single copy of the wild-type *SNCA* locus, associated with a dose-dependent gain of toxic function, such that patients harboring *SNCA* triplication exhibit earlier PD onset, more aggressive clinical severity, and faster disease progression^7^.

Despite the genetic evidence mentioned above, the precise mechanisms underlying how αSyn contributes to neuronal dysfunction and death in synucleinopathy remain elusive. The resemblance of αSyn to lipid-binding proteins has long been recognized^8^, and subsequent investigations have confirmed its ability to bind lipids, especially phospholipids and fatty acids^9^. While most of this highly expressed brain protein is cytosolic, a fraction of αSyn is bound to various lipid membranes, including synaptic vesicles and plasma membranes^9^. As highlighted by Musteikyté et al.^10^, the interaction between αSyn and lipid membranes is considered crucial for its biological function, and plays a significant role in the abnormal processes associated with αSyn aggregation and toxicity. Changes in membrane physical properties and chemical composition promote the aggregation of αSyn into toxic amyloid fibrils, while aggregated αSyn species bind to lipid membranes, compromising their integrity^10^. However, the precise mechanisms underlying this bidirectional neurotoxic scenario of αSyn-lipid membrane interaction have not yet been fully elucidated.

Our published data indicate that αSyn is also recruited to membrane domains localized in the endoplasmic reticulum (ER) in close apposition to mitochondria^11^, known as mitochondrial-associated ER membranes (MAMs). We believe this observation is highly relevant to the question of αSyn-lipid membrane crosstalk, as MAMs are transient lipid raft domains within the ER where key regulatory lipid enzymes converge to control membrane homeostasis^12^. Supporting this view, we found that cell lines stably expressing αSyn mutations display alterations in their lipidome^13^ and in MAM-related lipid metabolism pathways^11^.

To expand our work on αSyn and lipid metabolism in synucleinopathy and MAM, we began the present study by defining the lipidomic profiles of selected postmortem brain regions of PD patients. These analyses showed three main changes that differentiate the lipid profile of the severely affected brain region substantia nigra pars compacta (SNpc) in PD patients from both less affected brain regions in PD and the unaffected SNpc in non-PD controls: (i) elevated levels of cholesterol esters (CEs), (ii) reduced levels of specific ceramide (Cer) species, and (iii) the presence of specific phosphatidylcholine (PC) and phosphatidylserine (PS) species bound to long-chain and unsaturated fatty acids (FAs) that were not present in other PD brain regions or control SNpc. These findings suggest that lipid metabolism changes in PD are specific to certain regions, with the SNpc displaying unique lipid profiles compared to other brain regions affected by PD and control samples. To determine whether the observed lipid changes are specific to PD, we also performed lipidomic analysis in MSA as an example of another synucleinopathy. These additional analyses revealed similarities in lipid alterations between MSA and PD samples. However, the changes in lipid species were fewer and of lesser magnitude in MSA compared to PD, suggesting that several lipid alterations appear to be shared by PD and MSA, while others may be specific to vulnerable areas in PD.

In the second part of this study, we turned our attention to a simplified system provided by human induced pluripotent stem cell (iPSC)-derived midbrain neurons to elucidate the cell-autonomous mechanisms leading to the lipid alterations identified in postmortem tissues. These investigations revealed that iPSC-derived neurons harboring *SNCA* duplication recapitulated some of the lipidome changes observed in SNpc samples from PD cases. They also indicated that αSyn plays a physiological role in modulating lipid enzymes through its localization to MAM and that increased levels of αSyn interfere with this function, leading to defects in lipid homeostasis. Our findings indicate that αSyn pathology is associated with disrupted lipid homeostasis, potentially contributing to the susceptibility of specific brain regions in synucleinopathy. These insights carry important therapeutic implications.

## Results

### Altered lipidome in the postmortem PD brain

Studies conducted by us and others have reported alterations in lipid profiles in biofluids obtained from individuals with synucleinopathy compared with those from control individuals^13–18^. However, the molecular mechanisms underlying lipid changes associated with αSyn pathology and the differential susceptibility of brain regions in synucleinopathy remain unclear. In the present study, we aimed to address these questions by conducting an unbiased lipidomics analysis of postmortem samples from different brain regions obtained from 16 patients with PD and 14 age-matched control individuals (Supplementary Table 1). Because the SNpc has been identified as a primary site of neuropathological changes in PD^19^, we specifically compared the lipid profiles of the SNpc between patients with PD and non-PD controls. In addition to the SNpc, we also analyzed three other brain regions from the same cohort of PD donors, the ventral tegmental area (VTA), the substantia innominata (SI), and the rostral hypothalamus (Hypo). These regions provide a range of neurodegeneration, with the greatest degree of neuronal loss in the SNpc, followed by the VTA, the SI, and the lowest degree of neuronal loss in the rostral Hypo^20–25^. All collected samples were promptly flash-frozen in the presence of antioxidants (butylated hydroxytoluene) and subsequently processed for lipidomics analysis using ultra-high performance liquid chromatography coupled with tandem mass spectrometry, as previously described^13^. Lipids were extracted from 100 µg of tissue from each sample via the chloroform–methanol extraction, followed by a modified Bligh and Dyer protocol.

Triplicate aliquots were analyzed for each sample, allowing us to detect more than 500 lipid species belonging to 31 different classes (Supplementary Table 2). Using spiked internal standards with known concentrations, we determined the concentration of each lipid species. We applied the normalization using the optimal selection of multiple internal standards method^26^ and conducted the Principal Component Analysis to identify samples outside of the 95% confidence interval, which we classified as outliers (Supplementary Fig. 1). Subsequently, we compared the lipidome results from all brain areas under study by one-way ANOVA and plotted *p*-values for each species in a 3D volcano graph. The data for subset groups were reduced to a 2D polar coordinate system, as explained^27^ (Supplementary Fig. 2A). The 3D volcano plots helped us visualize that the groups display significant differences in their lipid composition. In particular, our data revealed some significant changes in the levels of specific lipids of CEs and diacylglycerides (DGs) especially those bound to oleic acid (CE 18.1 and DG 38.1), as well as various phospholipids such as those PC species bound to saturated FAs (PC 32.0) and those containing arachidonic acid (PC 38.4) (Supplementary Fig. 2A).

To reduce biases and increase confidence that our findings are not artifacts of a specific analytical methodology, we next applied an orthogonal approach using a Random Forest (RF) machine learning classifier to provide an independent and complementary perspective on our data, as previously done^16^. This approach revealed several lipid classes and species capable of discriminating between PD and control SNpc as well as SNpc and other brain areas (VTA, Hypo and SI) from the same PD cases (Table 1). The RF analysis identified that alterations in CEs, (CE 18:1, CE 20:3 and CE 22:6) and phosphatidylinositol (PI) and phosphatidylethanolamine plasmalogen (PEp) species with polyunsaturated acyl chains (PI 40:5 and PEp 36:4), are critical discriminatory variables among brain regions from the same cases and control group.

**Table 1:** Lipid alterations in postmortem PD brains

Donors (Brain region)	Lipid Groups	Lipid species
PD (SNpc v VTA)	Sterols	CE 18:1
		CE 20:3
		CE 22:6
	Phospholipids	PC 40:7
		PC 42:5
		PS 36:2
PD vs Controls (SNpc)	Sterols	CE 18:1
		CE 20:3
		CE 22:6
	Sphingolipids	Cer 20:0
		Cer 20:1
		Cer 22:0
	Phospholipids	PC 32:0
		PC 40:7
		PC 42:5
		PC 42:6
		PS 36:2
		PS 38:5
		PEp 36:3
		PEp 36:4

Lipid species identified by random forest (RF) classification can discriminate within PD brains differentially susceptible brain regions (substantia nigra pars compacta (SNpc) vs ventral tegmental area (VTA) (upper row) and between PD brains and control brains at the SNpc (lower row).

*SNpc* substantia nigra pars compacta*, VTA* ventral tegmental area*, CE* cholesteryl esters*, PC* phosphatidylcholine*, PS* phosphatidylserine*, Cer* ceramide*, PEp* plasmalogen phosphatidylethanolamine.

Other polyunsaturated phospholipids belonging to PE and PEp classes (PEp 36:3, PEp 36:5, PEp 38:3, PEp 38:2, PE 36:2, PE 38:2) were also found to be specifically altered in SNpc from PD, but only when compared to controls. Additionally, two Cer species bound to C22 FAs (Cer 22:0 and Cer 22:1) were classified as discriminatory variables able to distinguish between PD vs. control SNpc.

The minimal depth of the RF algorithm shows the distance between the root and the decision nodes using the particular lipid species in the decision trees for the classification. Higher frequencies at shorter distance would indicate that some lipid species are more effective at classifying the different groups. Our results suggest that, although at lower frequencies, defects in the levels of DG species, such as DG 38:1 or DG 38:3, are phenotypes unique to PD SNpc compared to controls (Supplementary Fig. 2B).

**Fig. 1:**
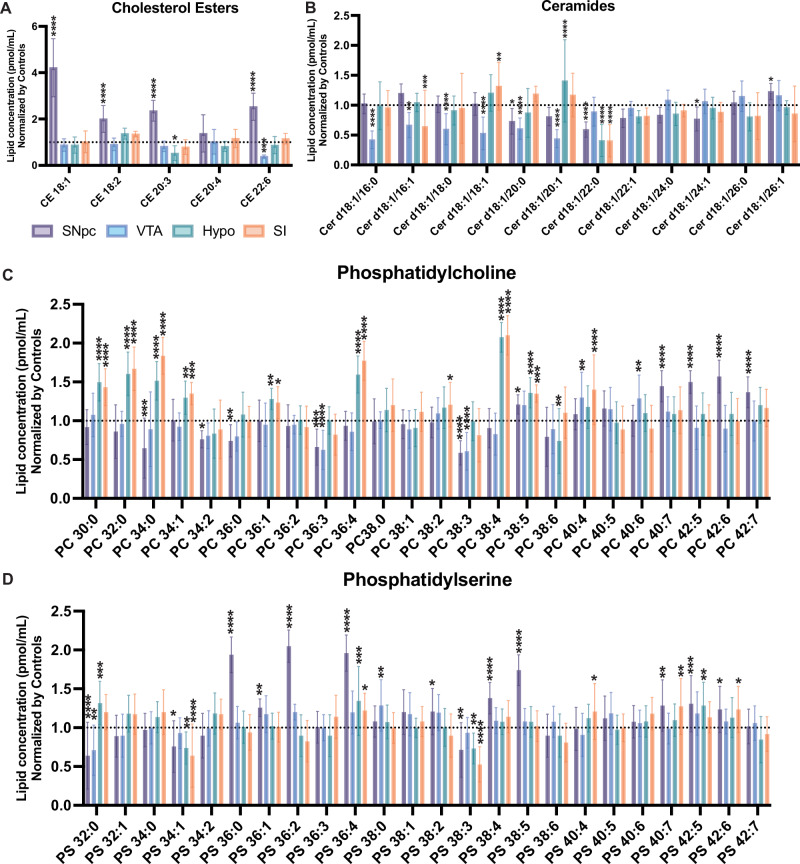
Lipid alterations in the postmortem Parkinson’s disease (PD). Relative concentrations of the indicate lipid species of: **A** esterified cholesterol (CE) (**B**) ceramide (Cer), **C** phosphatidylcholine (PC) and **D** phosphatidylserine (PS) in homogenates of different brain regions: substantia nigra pars compacta (SNpc), ventral tegmental area (VTA), hypothalamus (Hypo), substantia innominata (SI) of PD postmortem donors (*n* = 16 for each brain region). Lipid species were normalized to concentrations in Control samples (*n* = 14) (indicated by dotted lines). Data are means ± SD of independent biological replicates (n) analyzed by a two-way ANOVA. (**A**) CE: Interaction (F_(16,365)_ = 22.84; ****p < 0.0001), **B** Cer: Interaction (F_(44,876)_ = 12.01; ****p < 0.0001), (**C**) PC: Interaction (F_(92,1752)_ = 14.61; ****p < 0.0001), (**D**) PS: Interaction (F_(92,1752)_ = 11.98; ****p < 0.0001). For post-hoc analyses (**A–D**), Dunnett’s multiple comparison test with a single pooled variance was used for each brain region vs Controls. *p < 0.05, ** *p* < 0.01, ***p < 0.001 ****p < 0.0001.

To validate these data, we also compared the levels of various lipid classes and species between PD and control samples and calculated fold-change values. In agreement with our RF results, SNpc samples from patients with PD exhibited higher levels of specific CE species (Fig. 1A) than SNpc samples from controls. Our analysis also revealed notable reductions in the levels of various abundant Cer species in the SNpc of patients with PD relative to the levels observed in other areas of the brain and control SNpc, particularly those linked to long-chain FAs (Fig. 1B). Furthermore, we found unique alterations in the levels of major phospholipids in PD SNpc compared to the rest of the PD regions under study and SNpc from controls. Namely, our results show that PD SNpc samples displayed higher levels of PC (Fig. 1) and PS (Fig. 1D) species bound to long-chain and unsaturated FAs that were not present in the other PD brain regions or control SNpc, whereas elevated levels of PE and PEp species bound to long-chain and unsaturated FAs were found in other brain regions that was not the SNpc (Supplementary Fig. 2C). Thus, our analyses reveal alterations in three primary classes of lipids: sterols, phospholipids, and sphingolipids. These changes appear region-specific, with the SNpc in PD exhibiting distinct lipid profiles compared to other PD-affected brain regions and control samples.

We next asked whether the lipid changes reported above are specific to PD or shared by other synucleinopathies. Accordingly, we analyzed the lipidome of striatum samples from MSA cases (Supplementary Table 3), since it is the primary site of neuropathological changes in MSA^19^, and compared it to our data from PD and non-PD controls. These analyses revealed that the striatum in MSA has alterations in some of the CE species like those detected in the SNpc of PD cases (Supplementary Fig. 3A, B). In addition, we were also able to find increases in PS species bound to long-chain and unsaturated FAs (e.g., PS 38:4, PS 38:5) like those found in PD SNpc samples. However, the magnitude and extent of the lipid changes in the MSA striata were not as profound as those found in PD SNpc samples and did not replicate the alterations in Cer, and PC levels seen in the latter (Supplementary Fig. 3A, B). Thus, the partial similarity of lipid alterations between PD SNpc and MSA striatum suggests that some observed changes detected in affected brain areas are shared between these two distinct synucleinopathies. In contrast, others, such as alterations in Cer, and PC seem specific to PD, SNpc, or both.

### Lipid alterations in human iPSC-derived neurons are associated with αSyn gene-dosage

To shed light on the mechanisms driving the observed lipid alterations in the brains of patients with synucleinopathy, we used human iPSC-derived neurons as a simplified model system that would be more suitable for biochemical and molecular investigations. Because alterations in αSyn expression are strongly implicated in PD pathogenesis^28^, we sought to assess the impact of αSyn dosage on neuronal lipid metabolism.

Thus, cultured iPSC-derived neurons were used to conduct the same lipidomics analyses applied to brain samples using iPSC lines expressing varying levels of αSyn, including iPSCs in which αSyn expression was knocked out (αSyn-KO), a line expressing endogenous αSyn levels (αSyn-NL), and a line expressing 1.5× endogenous levels (αSyn-Duplication). These human fibroblast-reprogrammed iPSC lines were obtained from different individuals, and their characterizations are detailed in Supplementary Table 4, and previous publications^29–33^. As previously described, iPSCs were first differentiated into neural precursor cells, which were used to derive human neurons, as reported previously^29–31,33–35^. After 30 days in vitro (DIV30) under directed neuronal differentiation conditions, >90% of neural precursor cells from a minimum of 4 independent directed differentiations into neurons, as evidenced by the expression of the neuronal markers Tuj1 and Map2. Among these neurons, 20–30% expressed dopaminergic markers, including tyrosine hydroxylase (TH) and dopamine transporter (DAT), and were characterized by a ventral midbrain identity, as evidenced by the expression of FoxA2, in keeping with previous studies^30,36^ (Supplementary Fig. 4A–C). Using immunoblot analysis, we confirmed that iPSC-derived αSyn-Duplication neurons expressed 50% more αSyn than age-matched αSyn-NL neurons, and that iPSC-derived αSyn-KO neurons did not express αSyn (Supplementary Fig. 4D)^35,37^. Based on a minimum of four independent directed differentiations, no significant differences were observed in differentiation efficiencies or survival rates across the various iPSC-derived neuronal lines.

**Fig. 2:**
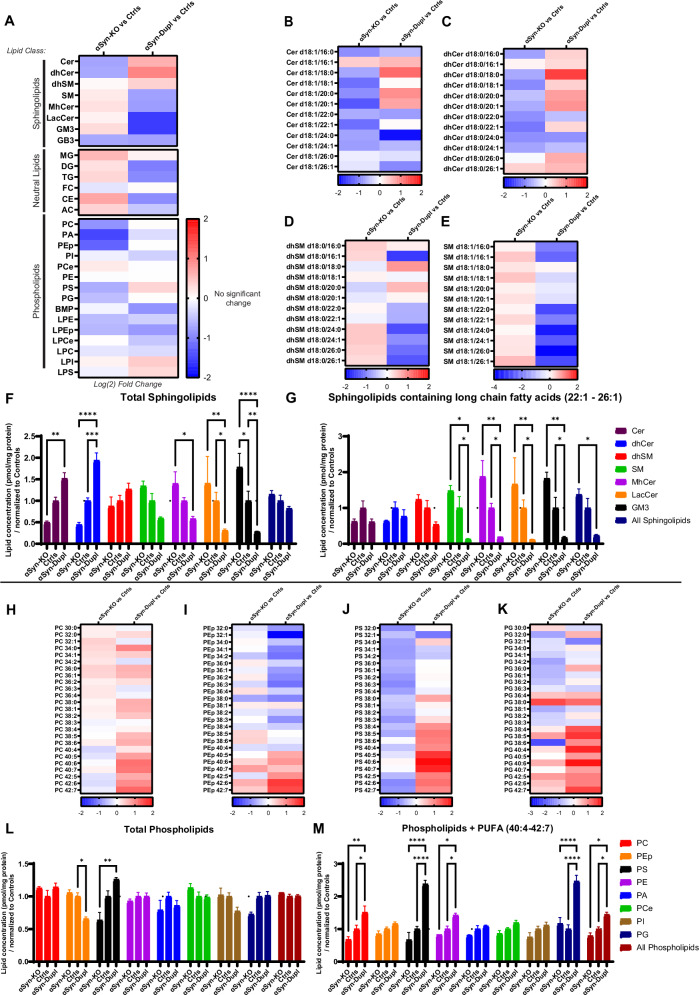
Lipid alterations in patient-derived neurons expressing differing alpha-synuclein (αSyn) doses. Lipidomic heat maps showing Log(2) fold changes (**A**) all lipid groups and selected individual lipid species of: **B** Ceramide (Cer), **C** dihydroceramide (dhCer), **D** dihydrosphingomyelin (dhSM) and **E** sphingomyelin (SM) in induced pluripotent stem cell (iPSC)-derived neurons carrying either αSyn knock out (αSyn-KO) or αSyn duplication (αSyn-Dupl), normalized to age-matched neurons expressing endogenous αSyn levels (Ctrls) after 30 days of directed differentiation. **F** Relative concentrations of total sphingolipids and **G** sphingolipids containing long-chain fatty acids (22:1 to 26:1) are presented. Lipidomic heat maps displaying Log (2) fold changes in selected phospholipids: **H** Phosphatidylcholine (PC), **I** Plasmalogen phosphatidylethanolamine (PEp), **J** Phosphatidylserine (PS), **K** Phosphatidylglycerol (PG). Relative concentrations of **L** total phospholipids and **M** phospholipids containing polyunsaturated fatty acids (PUFAs, 40:4 to 42:7) are presented. All data are normalized to the concentrations in Ctrl samples. Data are presented as the mean ± SEM of at least 4 independent biological replicates (n) analyzed by an ordinary two-way analysis of variance ANOVA, with the interaction of cell line x lipid group given in: **F** Total sphingolipids: Interaction (F_(16,99)_ = 5.462; ****p < 0.0001), **G** Sphingolipids containing long-chain fatty acids: Interaction (F_(16,99)_ = 1_._522; *p* = 0.1068), Row Factor (cell line) (F_(2,99)_ = 19_._09; ****p < 0.0001); (**L**) Total phospholipids, Interaction (F_(18,110)_ = 2.485; **p_=_0.002); (**M**) Phospholipids containing long-chain fatty acids: Interaction (F_(18,110)_ = 5.215; ***p < 0.0001). For post-hoc analyses (**F**, **G** & **L**, **M**) a Tukey’s multiple comparison test was used with single pooled variance, *p < 0.05, **p < 0.01, ***p < 0.001 ****p < 0.0001.

Using these various cell lines, our lipidomic analysis revealed that αSyn-Duplication neurons exhibited substantial changes in sphingolipids compared to both αSyn-NL and αSyn-KO neurons. Indeed, we found increases in the overall levels of Cer and its saturated precursor dihydro-Cer (Fig. 2A, C, F), as well as in those of the dihydrosphingomyelin (dhSM) lipid class in αSyn-Duplication neurons compared to controls (Fig. 2D). However, when all the species from these sphingolipid classes were analyzed (Fig. 2E, F), we observed a marked decrease in those containing long-chain FAs in αSyn-Duplication neurons compared to controls (Fig. 2F, G). Moreover, complex sphingolipids, such as monohexosylceramide (MhCer) and lactosylceramide (LacCer), were reduced both in overall abundances and in the abundance of species containing long-chain FAs in αSyn-Duplication neurons compared with control neurons (Fig. 2F, G). In contrast, αSyn-KO neurons exhibited increases in the levels of these species (Fig. 2A, F–G, Supplementary Fig. 4E). A similar observation was made for GM3 (mono-sialodihexosyl-ganglioside) (Fig. 2A, F, G, Supplementary Fig. 4E). Concomitantly, αSyn-Duplication neurons displayed increases in sphingolipid species with shorter acyl chains, while αSyn-KO neurons presented the inverse phenotype (Fig. 2A, F, G and Supplementary Fig. 4E), suggesting that these lipid alterations are inversely correlated with the αSyn dosage.

As for phospholipids, αSyn-Duplication neurons displayed marked differences in this lipid class compared with control and αSyn-KO neurons (Fig. 2H–K and Supplementary Fig. 4F). Notably, we detected an increase in the concentration of PS (Fig. 2L) as well as all species of phospholipids bound to polyunsaturated fatty acids (PUFAs; 40:4 to 42:7) (Fig. 2M), including not only PS and PC but also phosphatidylethanolamine (PE) and phosphatidylglycerol (PG) (Fig. 2B). Deviations in the opposite direction were observed for these species in αSyn-KO neurons, suggesting a potential link between the levels of these lipid species and the αSyn dose. However, not all phospholipid species were increased as the concentration of PEp was decreased in αSyn-Duplication neurons compared with αSyn-NL neurons (Fig. 2I and L). Thus, our whole-cell lipidomic analysis of αSyn-Duplication neurons provided valuable insights into the alterations in both sphingolipids and phospholipids in connection to αSyn dosage. This underscores the value of our cell model in exploring the association between αSyn and lipid metabolism changes in synucleinopathy.

Given these findings, we next sought to compare the lipid changes observed in SNpc tissues with those in iPSC-Duplication samples. While no significant changes in sterols were detected in iPSC-Duplication neurons as seen in PD SNpc, both sample types exhibited similar alterations across several other lipid classes and species. As shown in the accompanying heatmap (Supplementary Fig. 5), both displayed elevations in specific PS and PC species bound to PUFAs, along with significant reductions in total SM levels and certain Cer species. Notably, none of the significant changes exhibited opposite directions between the two sample types.

In addition to evaluating lipid concentrations and their directional changes, we assessed the slope of change for each lipid species in relation to their respective controls, based on the extent of alteration observed across individual samples. Although the distinct nature of these samples precluded a quantitative correlation analysis (Supplementary Fig. 5), our results revealed remarkably similar lipid alterations in the species mentioned above. These findings suggest the presence of shared underlying mechanisms in both iPSC-derived cells and SNpc tissues.

### Analysis of subcellular fractions from cell models expressing endogenous αSyn

**Fig. 3:**
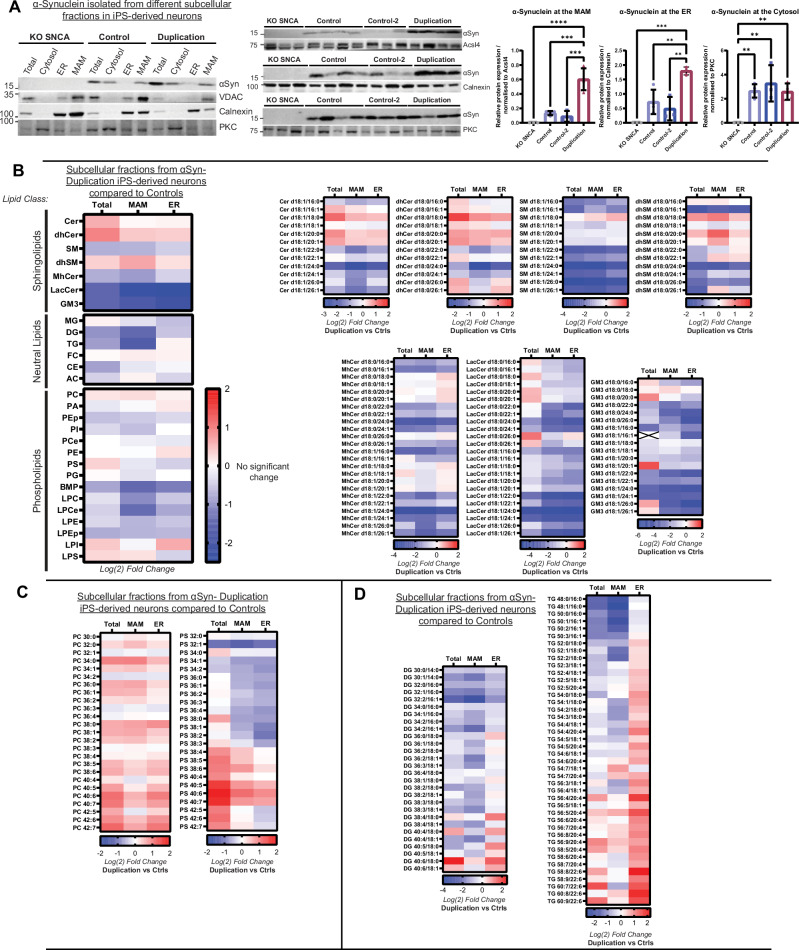
Lipid alterations in neurons with alpha-synuclein (αSyn) duplication across subcellular fractions. **A** Qualitative (left) and quantitative (right) assessments of monomeric αSyn distributions in the total unfractionated homogenate and in mitochondria-associated endoplasmic reticulum (ER) membrane (MAM), ER, and cytosol fractions from induced pluripotent stem cell (iPSC)-derived neurons carrying different αSyn levels after 30 days of directed differentiation, from a minimum of 3 independent directed differentiations. A semi-quantitative assessment of αSyn abundance was performed using protein immunoblots. Protein levels were normalized to the levels of long-chain fatty acid–CoA ligase 4 (Acsl4, MAM marker), calnexin (bulk ER marker), or protein kinase C (PKC, cytosolic marker). Data are presented as the mean ± SD of at least 3 independent biological replicates (n) analyzed by an one-way analysis of variance (ANOVA). The variation between the cell lines per fraction given, MAM: (F_(3,10)_ = 32.56 ****p < 0.0001); ER (F_(3,9)_ = 17.21 ***p = 0.0005); Cytosol (F_(3,10)_ = 9.424 **p = 0.0029). For post-hoc analyses a Tukey’s multiple comparison test was used with single pooled variance, *p < 0.05, **p < 0.01, ***p < 0.001 ****p < 0.0001. **B** Lipidomic heat maps displaying Log_2_ fold changes in groups and individual lipid species of sphingolipids; neutral lipids, and phospholipids in αSyn-Duplication iPSC-derived neurons compared with controls (Ctrls) in the total unfractionated homogenate and in MAM and ER fractions. In Ctrl neurons, alterations in sphingolipid species were consistent between MAM and ER fractions (right). **C** MAM and ER fractions show elevated phosphatidylcholine and phosphatidylserine concentrations, but a specific reduction in phosphatidylserine lipid species comprised of smaller-chained hydrocarbons is also observed. **D** Individual neutral lipids species across the total homogenate, MAM, and ER fractions highlight notable differences in diglyceride (DG) and triglyceride (TG) lipids between fractions.

The convergence of lipid metabolic pathways on MAM domains regulates cellular lipid homeostasis. Our published data indicate that αSyn can localize to MAM domains and that point mutations in *SNCA* or overexpression of wild-type αSyn disturb the regulation of MAM domains and MAM-associated cellular functions^11^. To confirm these findings in iPSC-derived neurons, we first performed subcellular fractionation as in^11^, using a minimum of 4 independent directed differentiations per cell line to assess αSyn content of purified ER, MAM, mitochondria, and cytosol by immunoblot (Fig. 3A, Supplementary Fig 4G) This experiment confirms that αSyn localizes to the MAM and cytosolic fractions in αSyn-NL iPSC-derived neurons, with limited localization in ER fractions (Fig. 3). In the total non-fractionated homogenate, the αSyn-Duplication line expresses 1.5× the level of αSyn detected the αSyn-NL line, consistent with the duplication phenotype (Supplementary Fig. 4D); however, the relative quantification of subcellular fractions from αSyn-Duplication neurons revealed the relative enrichment of αSyn in the MAM and ER fractions, with levels 3× and 4× those found in the respective fractions from αSyn-NL neurons. These findings indicate that the αSyn localization is altered when the concentration increases above endogenous physiological levels. This leads to the selective accumulation and association of αSyn at MAM and ER domains (Fig. 3A).

The MAM domain is a transient lipid raft induced by the clustering of cholesterol, sphingomyelin (SM), and saturated phospholipids^38^ that modulates specific protein subsets^39^. Alterations in the lipid compositions of MAM domains impair the enzymatic activities that localize and are regulated in these regions^40,41^. Thus, next, we applied our lipidomics analysis to MAM and ER fractions from αSyn-Duplication and αSyn-NL neurons to expand our analysis of lipid compositions in these cell lines. Consistent with our whole cell lipidomic analysis, our data did not reveal any alterations in sterols in MAM and ER fractions among genotypes but both in sphingolipids and phospholipids. Indeed, in the case of sphingolipids, we found that the levels of SM and complex sphingolipids were reduced in αSyn-Duplication neurons compared with αSyn-NL neurons, and a reciprocal relationship between Cer and SM was noted in the total homogenates and in ER and MAM fractions (Fig. 3B, Supplementary Fig. 6A). Concomitantly, the overall concentration of dihydro-SM species increased in MAM fractions from αSyn-Duplication neurons compared with MAM fractions from αSyn-NL neurons (Fig. 3B). Further lipidomics analysis indicated an imbalance in sphingolipid species in αSyn-Duplication neurons compared with αSyn-NL neurons, characterized by a marked decrease in species bound to long acyl chains and a slight increase in shorter and saturated species from the dihydro-SM, dihydro-Cer, and Cer classes (Fig. 3B). Interestingly, subcellular fractions from αSyn-KO neurons showed the opposite phenotype of those from αSyn-Duplication neurons, further supporting the potential contribution of αSyn levels to the modulation of sphingolipid homeostasis (Supplementary Fig. 6A, B).

Regarding phospholipids, similar to the alterations observed in total homogenates, the MAM and ER fractions of αSyn-Duplication neurons displayed elevated levels of PC and PS species bound to PUFAs compared with those fractions from αSyn-NL neurons (Fig. 3C). Because PS is synthesized at MAM domains, the disparity between PS bound to long-chain PUFAs and PS bound to shorter-chain and more saturated fatty acyl chains was particularly striking, suggesting that the metabolism of PS is impaired at MAM domains in αSyn-Duplication neurons (Fig. 3C). As before, αSyn-KO neurons showed the inverse phenotype to the phenotype observed in αSyn-Duplication neurons, implying a positive relationship between αSyn dosage and PS metabolism (Supplementary Fig. 6C).

Although we observed reductions in the overall lipid species of iPSC-neuronal extracts from the αSyn-Duplication neurons in the total and MAM fraction (Supplementary Fig. 6B), we found striking lipid changes and high concentrations of certain DG and triglyceride (TG) species in MAM and ER fractions (Fig. 3D). These elevations were particularly notable in TG species containing oleic acid (C18:1) and long PUFAs, such as arachidonic acid (C20:4) and docosahexaenoic acid (DHA) (C22:6) were elevated in the ER and DG species that were reduced in the MAM. In contrast both DG and TG species containing SFAs, such as palmitic acid (C16:0) and stearic acid (C18:0) were specifically reduced at MAM domains (Supplementary Fig. 6D, E). These findings suggest that significant alterations in lipid species occur at the MAM/ER domains and that de novo TG synthesis takes place in the ER of αSyn-Duplication neurons.

Altogether, our lipidomics results reveal an association between high levels of αSyn and the disruption of membrane composition and the lipid milieu of ER and MAM fractions, which can help explain previously reported defects in MAM activities and ER–mitochondria crosstalk^11^.

### Alterations in the PS synthetic machinery parallel αSyn dosage in iPSC-derived neurons

**Fig. 4:**
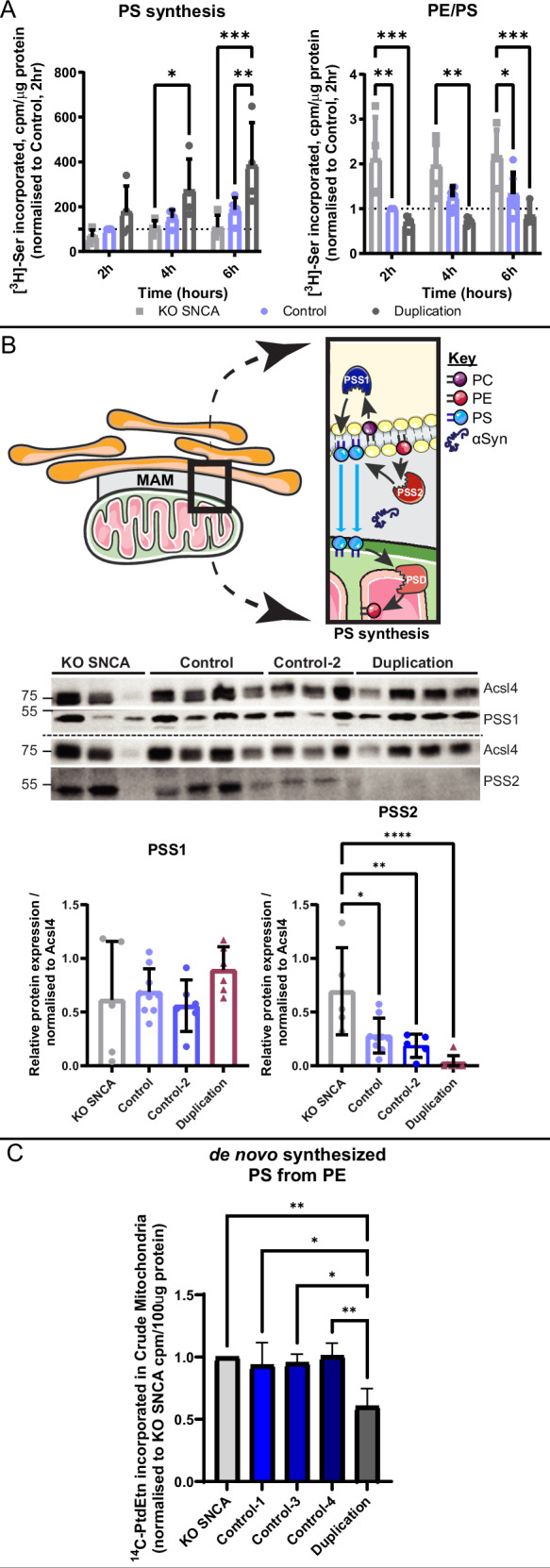
Different alpha-synuclein (αSyn) levels alter mitochondria-associated endoplasmic reticulum membrane (MAM) function. **A** Phospholipid synthesis in induced pluripotent stem cell (iPSC)-derived neurons. Quantification of de novo phosphatidylserine (PS) synthesis and the ratio phosphatidylethanolamine (PE) to PS (PE/PS) levels in patient-derived neurons after 30 days of directed differentiation, after incubation with ^3^H-Serine [^3^H-Ser] for the indicated times. Data were normalized to the Control cell line, and at least 4 independent differentiations were performed. A repeated measures two-way analysis of variance (ANOVA): PS synthesis: Column (Cell line) factor (F_(2,10)_ = 6.777; *p = 0.0138); Row (time) factor (F_(2,20) =_ 10.92; ***p = 0.0006); PE/PS: Cell line factor (F_(2,11)_ = 13.41; **p = 0.0011); time factor (F_(2,22) =_ 1.180; *p* = 0.3260). For post-hoc analyses a Tukey’s multiple comparison test was used with single pooled variance, *p < 0.05, **p < 0.01, ***p < 0.001 ****p < 0.0001. **B** Protein levels of PS synthase (PSS)1 and PSS2 at the MAM domain. Analysis of MAM fractions from iPSC-derived neurons probed for PSS1 and PSS2, with a representative image shown, adapted with permission according to CC BY 4.0 from ref. ^97^. Data are presented as the mean ± SD of at least 5 independent biological replicates (n) analyzed by an one-way ANOVA. The variation between the cell lines of the indicated proteins is given as: PSS1 (F_(4,28)_ = 1.259; *p* = 0.3094); PSS2: (F_(4,28)_ = 7_._741; ***p = 0.0002). Tukey’s multiple comparison was used for post hoc analysis with a single pooled variance. *p < 0.05, **p < 0.01, ***p < 0.001, ****p < 0.0001. **C** Assessment of PSS2 activity. Quantification of de novo PS synthesis from [^14^C]-PE in patient-derived neurons after 30 days of directed differentiation. After subcellular fractionation and quantification, 100 µg of protein isolated from the crude mitochondria was incubated with [^14^C]-PE for 45 minutes. Lipids were immediately extracted using the chloroform/methanol extraction method followed by a modified Bligh and Dyer protocol. A minimum of 3 independent differentiations per cell line were performed, and data were normalized to the αSyn-KO cell line. A repeated measure one-way ANOVA with assumed sphericity was performed with the variation between the cell lines given: (F _(4,14)_ = 7.893; **p = 0.0015). For post-hoc analyses a Tukey’s multiple comparison test was used with single pooled variance, *p < 0.05, **p < 0.01, ***p < 0.001 ****p < 0.0001.

We then examined the impact of αSyn dosage on MAM functionality, focusing on PS metabolism due to the aforementioned alterations in this lipid class. Accordingly, we monitored [^3^H]-PS formation and its decarboxylation to [^3^H]-PE by incubating our cell models with [^3^H]-serine, as previously described^11^, using a minimum of 4 independent directed differentiations per cell line. Our findings indicated that upon incubation with [^3^H]-serine, αSyn-Duplication neurons have a ~2-fold higher content of [^3^H]PS compared with αSyn-NL neurons and a ~4-fold higher content compared with αSyn-KO neurons (Fig. 4A). Conversely, upon incubation with [^3^H]-serine, we found a negative relationship between αSyn dosage and [^3^H]-PE content in the same cell models (Fig. 4A). These results suggest that αSyn-Duplication is associated with an impaired decarboxylation of PS into PE, albeit we cannot exclude that PS synthesis is also increased in these neurons.

The MAM domains have been identified as the subcellular locus for PS synthesis by the PS synthase (PSS) enzymes PSS1 and PSS2^42^. Thus, to study further the question of PS metabolism in αSyn-Duplication neurons, we conducted immunoblot analysis to examine the levels of PSS1 and PSS2, which use PC and PE, respectively, as substrates^43^ (Fig. 4B). MAM fractions isolated from iPSC-derived neurons at DIV30 revealed an inverse relationship between PSS2 immunopositivity and αSyn expression; however, no differences in PSS1 immunopositivity were observed among αSyn-KO, αSyn-NL, and αSyn-Duplication neurons (Fig. 4B). Similarly, no differences in PS decarboxylase (PISD) immunopositivity were observed among αSyn-KO, αSyn-NL, and αSyn-Duplication neurons (Supplementary Fig. 7). We next used [^14^C]-PE to assess PSS enzymatic activity in isolated crude mitochondria preparations obtained from αSyn-KO, αSyn-NL, and αSyn-Duplication neurons. In keeping with the immunoblot findings of reduced PSS2 levels in αSyn-Duplication neurons, we observed a reduction in PS synthesis from [^14^C]-PE in αSyn-Duplication neurons compared with αSyn-NL and αSyn-KO neurons (Fig. 4C). Of note, since [^14^C]-PE can be used by both PSS1 and PSS2, the result of this enzymatic assay supports our PSS2 Western blot findings, but, by itself, does not indicate whether the reduced PS synthesis is due to a catalytic defect in either or both enzymes.

## Discussion

In this study, we investigated lipid alterations in PD, a prototypical synucleinopathy, by analyzing postmortem brain samples and comparing them to control samples as well as MSA, an alternative model of synucleinopathy. Using lipid profiling and RF classification, we previously utilized in human clonal cell lines expressing mutant αSyn-A53T^13^, we identified region-specific changes in sterols, phospholipids, and sphingolipids. Notably, the SNpc in PD exhibited distinct lipid profiles, particularly in CE, Cer, and polyunsaturated phospholipids. Several of these alterations, including in CEs, glycerolipids (e.g., TG), glycerophospholipids (e.g., PEp or PC), and sphingolipids (e.g., Cer) have been consistently observed in patients with PD^44^. To some extent, similar changes have also been reported in other neurodegenerative disorders^45^, suggesting a potential shared lipid metabolism dysregulation across neurodegenerative conditions.

There were also changes in certain CE species in the SNpc of PD cases compared to other PD brain regions and the SNpc of non-PD controls. Some of these CE species were also increased in striatum and cerebellar samples from MSA patients^46^. Interestingly, similar elevations in CE species have been observed in Huntington’s disease^47^ and Alzheimer’s disease cases^48^, indicating that alterations in these lipid species may have broader relevance beyond synucleinopathy. These increases may reflect neuroinflammation, as cholesterol esterification is stimulated by microglia activation during neurodegeneration. In turn, these changes allow microglia to adjust their membranes to respond to specific pathogens for phagocytosis or clonal expansion during inflammation. Thus, the observed elevations in certain CE species in whole tissue extracts may act as indicators of pro-inflammatory conditions in the regions most affected by synucleinopathies and other neurodegenerative disorders and underscores the critical interplay between lipid metabolism and inflammation in these diseases.

At the brain tissue level, several lipid alterations are noteworthy, as they may either result from or contribute to pathogenic mechanisms in PD and related conditions, although their actual pathogenic significance, at this stage, remains speculative. SNpc exhibited a significant increase in PC species bound to PUFAs, the most abundant class of phospholipids. Notably, this elevation in PC-PUFAs, along with other phospholipids such as PGs and plasmalogen forms of PEs, was also observed in iPSC-derived neurons. Although the molecular mechanisms underlying the PC-PUFAs accumulation in postmortem brain tissues and cell models of synucleinopathies remain unclear, these lipids may exert both deleterious and protective effects. Increased polyunsaturation has been shown to promote αSyn aggregation^49^, and inhibition of stearoyl-CoA desaturase (SCD1), which reduces polyunsaturation, mitigates αSyn pathology^50,51^, supporting a harmful role of PC-PUFAs in synucleinopathies. Additionally, PC-PUFAs, as a distinct lipid class, contribute to ferroptosis by triggering the production of reactive species in mitochondria and promoting lipid peroxidation in the ER^52^. Moreover, this elevation may alter membrane fluidity^53^, which is essential for a host of cellular functions, and may promote neuroinflammation precursors by stimulating the synthesis of inflammatory mediators such as prostaglandins and leukotrienes^54^. Conversely, PC-PUFAs, particularly DHA, activate the Akt signaling pathway, promoting neuronal survival and differentiation^55^. Moreover, elevated PC-DHA levels have been associated with approximately a 50% reduction in the risk of developing all-cause dementia^56^. Thus, PC-PUFAs may play a dual role, contributing to both pathological and potentially beneficial effects, which are not mutually exclusive. These effects may likely contribute to a combination of cell-autonomous and non-cell-autonomous mechanisms in the pathogenesis of PD and related conditions.

Alterations in PE species were also notable. In our study, we observed reductions in specific PE species, such as PE 38:3, in the SNpc and VTA compared to other brain regions, with similar changes identified in iPSC-derived neuronal cultures. While the significance of these changes requires further investigation, the observed PE defects may arise from the disruption of MAMs and their crosstalk with mitochondria, as discussed below. Alternatively, since PEs and indeed PS are essential components of the autophagosome machinery, these lipidome alterations could reflect impairments in autophagosome formation, which occurs at the MAM or autophagy flux. This is particularly relevant to PD and related conditions, given that αSyn has been reported to disrupt chaperone-mediated autophagy^57^, and mutations in Parkin and PINK1—known genetic causes of PD—play critical roles in mitochondrial macro-autophagy^58^. Based on our findings, we propose that, in addition to these established determinants of impaired autophagy in synucleinopathies, lipid dyshomeostasis, particularly αSyn-related alterations in PE species, may serve as an additional contributor to autophagy dysfunction in PD and related conditions.

We also observed reductions in sphingolipid species bound to long-chain FAs in PD cases. These deficiencies may impair the synthesis and abundance of complex sphingolipids, such as gangliosides. Notably, mutations in αSyn have been associated with reductions in gangliosides like GM1, whose supplementation has been shown to mitigate αSyn burden and alleviate motor deficits in disease models^59^. The synthesis of complex sphingolipids, including gangliosides, relies on communication between the ER and the Golgi apparatus. Interestingly, αSyn has been implicated not only in ER-mitochondria crosstalk, but also in regulating vesicular transport between ER and Golgi membranes. Given the role of these inter-organelle contact sites in lipid homeostasis, it is plausible that αSyn mutations impair its ability to localize and regulate such contact sites. This dysfunction may lead to longitudinal alterations in the lipidome of affected tissues, which could serve as biomarkers for monitoring disease risk and progression in the context of dementia and neurodegeneration.

In PD cases, SNpc and, to a lesser extent, striatum samples from MSA patients showed marked elevations in specific species of PS, which were also observed in the iPSC-derived neurons carrying the pathogenic αSyn-Duplication. More strikingly, αSyn levels paralleled the de novo synthesis and abundance of PS in iPSC-derived neurons, which was associated with reduced PSS2 levels at MAM domains and PSS activity in crude mitochondrial fractions. These findings led us to use AlphaFold 3^60^ to explore potential protein-protein interactions between αSyn and PSS enzymes, as no such interactions were identified through literature mining. Notably, AlphaFold 3 did not predict a direct interaction between αSyn and PSS enzymes. However, we recognize the challenges in modeling transient and weak interactions^61,62^, which cannot be fully excluded. Despite this possible limitation, we instead hypothesize that αSyn, by altering MAM lipid composition, impacts PSS activity. This proposed scenario aligns with our reported association of αSyn and MAM lipid changes, as well as the role of lipids such as PS in regulating PSS enzymes^63^. Thus, we suggest that αSyn in PD disrupts this function, leading to substantial defects in lipid homeostasis and membrane functionality.

Moreover, our lipidomics data from isolated MAM fractions suggest that αSyn pathology may interfere with ER membrane reorganization, which is essential for MAM formation. This disruption could impair not only PSS1 and PSS2, but also other enzymes within the MAM domain. For instance, changes in the lipid milieu after MAM formation activates phospholipid synthesis by enzymes such as PSS1 and PSS2 as well as FA acylation by long-chain fatty-acid–CoA ligases (Acsls)^43,64^. These MAM-resident enzymes activate saturated FAs, monosaturated FAs (Acsl1), and PUFAs (Acsl4, Acsl5 and Acsl6), a process vital for maintaining lipid homeostasis and supporting cellular functions related to lipid metabolism. The simultaneous regulation of these enzymes modulates the degree of phospholipid saturation and, thus, membrane fluidity and permeability. Therefore, the αSyn-related alterations in the formation and regulation of MAM domains may be the cause of the modifications in phospholipid synthesis, desaturation, and membrane fluidity, discussed above. In particular, evidence supports the existence of an interplay between αSyn and PS. αSyn binds to PS, with a preference for PS comprised of PUFAs^65^. The accumulation of insoluble αSyn in PD parallels PS abundance^66^ and alterations in de novo PS synthesis regulation.

Worth noting are studies comparing the activities of PSS enzymes in the brains of patients with PD and healthy controls have revealed reduced PSS enzymatic activity levels in the SNpc compared to the putamen and cortical regions in control brains^67^. However, contrary to our iPSC-derived results, patients with PD were reported to show increased PSS activity in the SNpc^67^. Since there is a profound loss of neurons associated with gliosis in the SNpc of PD^68^, this discrepancy may stem primarily from differences in the cellular landscape between the SNpc in PD and iPSCs, as at least PSS1 activity is more enriched in astrocytes than in neurons^69^. Interestingly, the activities of the rate-limiting enzymes involved in the de novo PC and PE synthesis through the Kennedy pathway (PCCT and PECT, respectively) also increase in the PD SNpc compared with the rates in healthy controls^67^. Furthermore, a meta-analysis of differentially expressed genes in patients with PD identified the downregulation of PSS1 in the SNpc^70^, suggesting that although dysregulated PS is involved in pathogenesis, the cell may compensate for phospholipid defects through multiple mechanisms. As reported by us and others, data suggest that these PS alterations are deeply associated with αSyn pathology and neurological deficits^71–73^, in agreement with previous observations^74–76^. On the other hand, these data do not clarify whether these phospholipid alterations occur in all cell types in most affected brain areas or just in populations with higher levels of αSyn aggregates.

One key pathological feature of neurodegenerative disorders, including synucleinopathies, is the differential susceptibility of subpopulations of neurons. The reason why, in disorders like PD, some dopaminergic neurons degenerate while others remain relatively unaffected is still an enigma. However, this study suggests that changes in specific lipids may offer an intriguing explanation. For instance, could it be that elevated levels of PS species bound to PUFAs in neurons and other brain cells modulate vulnerability to cell death mechanisms? At the plasma membrane, the loss of PS symmetry and the presence of extracellular-facing PS on the plasma membrane have been identified in the brains of patients with both AD and mild cognitive impairment^77^. Moreover, PS externalization is an “eat-me” signal for immune cells, including T cells, which designates the cell for immune attack and serves as an early indicator of apoptosis. In diseases associated with αSyn accumulation, the activation of microglia, T-cell ingress, and release of pro-inflammatory cytokines are all observed at the respective sites of neurodegeneration, suggesting that αSyn defects contribute to PS via impacts on MAM regulation, predisposing vulnerable cells to immune attack and death during disease progression.

Defects in sphingolipid regulation, caused by mutations in *GBA1*, have also been linked to increases in αSyn aggregation^78^. Altogether, these data reinforce the notion of a bidirectional interplay between αSyn regulation and sphingolipid homeostasis^79^, which may explain why *GBA1* mutations in the context of Gaucher disease are associated with increased PD risk. These findings align with the existence of a bidirectional regulation between αSyn and sphingolipids^80^, providing insights into the potential link between mutations in *GBA1* and *SNCA* that connect sphingolipid dyshomeostasis with increased PD risk. These sphingolipid alterations, however, were not statistically significant in striatum or cerebellar samples from MSA cases.

The modulation of enzymes involved in the synthesis and acylation of TGs, such as the acyl-CoA: DG acyltransferase 2 (DGAT2), also takes place at the MAM^81^. Interestingly, we identified higher levels of specific TG species exclusively in the ER of αSyn-Duplication. Considering our data, we posit that elevated TG levels in the ER could be a consequence of dysregulated MAM function and structure provoked by elevated αSyn. In turn, αSyn affinity for TG-rich membranes can explain the high levels of αSyn observed in ER fractions isolated from αSyn-Duplication neurons. The enrichment and aggregation of αSyn has also been associated with ER fragmentation and impaired function^82^.

Among the different TG species, those containing oleic acid and PUFAs were remarkably increased in αSyn-Duplication neurons, which agrees with the elevations in this type of TG species observed in sera from PD cases with the pathogenic αSyn-A53T mutation^82^. Notably, other studies have reported that blocking the oleic acid–generating enzyme, SCD1, which is also located at the MAM domain, rescues αSyn-mediated toxicity in human neurons and mice carrying another structurally altered pathogenic point mutation^51,83^.

In summary, our study provides a novel framework for understanding the role of αSyn in lipid metabolism at MAM, the disruption of which may contribute to the development of PD. Moreover, our data clarify the source of the well-known lipid alterations in patients with PD associated with αSyn mutations. In addition, our approach could reveal new disease biomarkers that may be developed into a new tool for determining PD risk, improving the accuracy of PD diagnoses, and predicting PD progression, mainly if analyzed jointly with other PD hallmarks.

## Methods

### Cell lines

The iPSC lines used in this study are shown in Supplementary Table 4; culture conditions have been previously described^32^. The generation of the αSyn-KO line using the CRISPR-Cas9 system was previously described^30,37^. For all experiments, iPSCs were directly differentiated to DIV30 via a neuronal precursor stage (NPCs) as previously performed^29–31,33–35^. Each independent replicate consisted of a separate directed differentiation from NPCs as previously described^29–31^.

### Subcellular fractionation of iPSC-derived neurons

A minimum of five confluent 15 cm^2^ plates of each cell line were subjected to directed differentiation. At DIV30, the plates were combined for each cell line and subjected to subcellular fractionation to obtain mitochondrial, ER, MAM, cytosolic, and crude mitochondrial (CM) fractionations, as previously described^84,85^. A minimum of four biological replicates were used per cell line.

### Protein immunoblotting

Protein immunoblotting was performed as previously described (30) using 4%–20% Tris-Glycine gels (Invitrogen; XP04205) to separate 20 µg/µL of denatured protein. A dry transfer was performed for all samples using the iBlot2 (Invitrogen; IB21001), and blots were probed using antibodies recognizing dopamine transporter (Sigma-Aldrich; D6944), vesicular monoamine transporter 2 (Santa Cruz; sc-374079), monoamine oxidase A (Proteintech Group; 10539-1-ap), tyrosine hydroxylase (Millipore; AB152), αSyn (C-42) (BD Transduction Labs; 610786), β-actin (Sigma-Aldrich; A5441), Erp72 (Cell Signaling Technology; D70D12), ATP5A1 (Invitrogen; 459240), ERLIN-2 (Cell Signaling Technology; 2959S), protein kinase C (Sigma-Aldrich; P5704), ACSL4 (Abgent; AP2536b), PSS1 (Abcam; Ab157222), PSS2 (Abcam; Ab183504) and PISD (Origene, TA807336). Membranes were washed in phosphate-buffered saline with 0.1% Tween 20 and probed with horseradish peroxidase-labeled anti-rabbit (Amersham; NA934V) or anti-mouse (Amersham; NA931V) secondary antibodies. The target bands were developed by enhanced chemiluminescence detection reagents (ThermoFisher; 34095 & 34580) and detected on the iBright 1500 Imaging System (ThermoFisher; A44114). Densitometry was performed using Fiji software^86^, and protein quantities were normalized where stated.

### Phosphatidylserine biosynthesis

De novo phosphatidylserine synthesis was performed on iPSC-derived neurons at DIV30 following directed differentiation, as previously described^11,84^.

### Phosphatidylserine synthase enzymatic activities

A minimum of three confluent 15 cm^2^ plates per cell line were subjected to directed differentiation. At DIV30, plates were combined for each cell line and subjected to subcellular fractionation to obtain CM, ER, and cytosolic fractions, as previously described^84,85^. A minimum of three biological replicates were used per cell line. Each subcellular fraction was quantified using the BCA assay according to the manufacturer’s instructions, and 100 µM of protein per fraction was incubated with 1 µCi PE, L-a-1-palmitoyl-2-arachidonyl [arachidonyl-1-^14^C] (American Radiolabeled Chemicals; 50-60 mCi/mmol) for 45 minutes at 37 ^o^C. The reaction was terminated by adding chloroform/methanol (2:1, v/v). Lipids were then extracted using the modified Bligh and Dyer method^87^, and thin-layer chromatography was performed as previously described^11,84^. Radiolabeled samples were read on a liquid scintillation counter (Perkin Elmer, Waltham, MA).

### Lipidomics

Lipidomics profiling was performed using UPLC–MS/MS^88,89^. The brain tissues and cell samples were processed and analyzed on two different platforms. The brain tissues were normalized by cutting out an equal block of 60 mm^3^ for all samples, then the modified Bligh and Dyer method^75^ was used for lipid extraction. The frozen brain blocks were sliced to the same thickness (5 mm), then punched out by using a hollow metallic cylinder (4 mm ID) to collect pellets of equal volume. The pellets were transferred into 2 mL Eppendorf tubes with 900 µL ice chilled chloroform/methanol (1:2; v/v) and were mechanically homogenized. The homogenates were vortexed for 15 seconds, then incubated in 4 °C on a mixer (300 rpm) for 1 hour. After agitation, 300 µL of ice‐cold chloroform and 250 µL of ice‐cold MQ water were added sequentially, followed by vortex for 15 seconds, and centrifugation at 9000 rpm for 2 min to separate the phases. Bottom organic phases were collected, and the aqueous phases were reextracted with 500 µL of chilled chloroform. Collected organic phases were dried in a vacuum concentrator and stored lyophilized at −80 °C.

For the lipidomics of the brain tissue, Samples were solubilized in 100 μL chloroform/methanol (1:1; v/v) and analyzed using a Waters Acquity UPLC I class coupled with a Waters Synapt G2‐Si (Waters Corp, Milford, MA). Lipids were separated in reverse phase using an Acquity UPLC HSS T3 1.8 μm column with the following conditions: mobile phase A (water:acetronitrile, 40:60, with 10 μM ammonium acetate and 0.1% acetic acid), B (water:acetonitrile:isopropanol: acetic acid, 5:10:85:0.1, with 10 μM ammonium acetate and 0.1% acetic acid); flow rate of 300 μL/min; injection volume of 5 μL; column temperature at 55 °C; 20% B for 1.5 min; linear change to 100% B over next 16.5 min; and maintained at 100% B for 3 min. Then, the gradient was reverted back to the initial state 20% B for 1 min, then held for the next 1 min at 20% B. Quality control (QC) samples were injected prior to, and after every 5 samples, to monitor the stability of the instrument. Samples were run under untargeted positive and negative electrospray ionization (ESI) modes in a data‐independent manner (MSE mode). The following ESI conditions were used: for positive, capillary at 2 kV, sampling cone at 35 V, source temperature at 100 °C, desolvation gas at 500 l/h, and nebulizer at 6.5 bar; and for negative, capillary at 2.2 kV, sampling cone at 40 V, source temperature at 80°C, desolvation gas at 500 l/h, and nebulizer at 6.5 bar. For lock mass correction, leucine enkephalin was used at 1 ng/mL in acetonitrile/water (1:1, v/v) with 0.1% formic acid and at a flow rate of 10 μL/min. The low collision energy was set to 4 eV, and the high collision energy was set between 25 and 40 eV for both positive and negative modes. Pooled samples were run at first and every after 6 samples as QC. Raw data were converted into ABF format using Reifycs Analysis Base File Converter, then used in MS‐Dial (ver. 4.9) for peak picking and retention time alignment using default settings. Lipid species were manually verified and named using Lipid Maps abbreviations. The intensities were initially normalized with the total ionic current. The corrected readings of identified species were exported into R (ver. 4.4.2) to do the batch correction using pooled samples as QC, then calculate the concentrations using the known concentration of spiked internal standards.

For the preparation of lipid extracts from total cell lysates and subcellular fractions. Equal levels of protein (100 µg) were used, that was determined using a BCA assay (see Methods). The data was given as mol% after normalization to the total lipid content. Relative molar amounts of lipid species in each sample were calculated based on multiple class-based internal standards (Avanti Polar Lipids, Alabaster, AL) using a modified Bligh and Dyer method^75^ .The data were then normalized to the sum of these molar contributions to obtain comparable relative contributions of each lipid species or class and expressed as mol % relative to the total amount of all measured lipid species^90^. The lipid extracts were spiked with appropriate internal standards using a modified Bligh and Dyer method^87^ and analyzed on a platform comprising an Agilent 1260 Infinity HPLC integrated to an Agilent 6490 A QQQ mass spectrometer controlled by Masshunter v7.0 (Agilent Technologies, Santa Clara, CA). Glycerophospholipids and sphingolipids were separated with normal-phase HPLC as described previously^91^, with a few modifications. An Agilent Zorbax Rx-Sil column (2.1 × 100 mm, 1.8 µm) maintained at 25 °C was used under the following conditions: mobile phase A (chloroform: methanol: ammonium hydroxide, 89.9:10:0.1, v/v) and mobile phase B (chloroform: methanol: water: ammonium hydroxide, 55:39:5.9:0.1, v/v); 95% A for 2 min, decreased linearly to 30% A over 18 min, and further decreased to 25% A over 3 min, before returning to 95% over 2 min and held for 6 min. Separation of sterols and glycerolipids was carried out on a reverse-phase Agilent Zorbax Eclipse XDB-C18 column (4.6 × 100 mm, 3.5 µm) using an isocratic mobile phase, chloroform, methanol, 0.1 M ammonium acetate (25:25:1) at a flow rate of 300 µL/min.

Quantification of lipid species was accomplished using multiple reaction monitoring transitions^91–93^ under both positive and negative ionization modes in conjunction with referencing of appropriate internal standards (lipid species abbreviations can be found in Supplementary Table 3): PA 14:0/14:0, PC 14:0/14:0, PE 14:0/14:0, PG 15:0/15:0, PI 17:0/20:4, PS 14:0/14:0, BMP 14:0/14:0, APG 14:0/14:0, LPC 17:0, LPE 14:0, LPI 13:0, Cer d18:1/17:0, SM d18:1/12:0, dhSM d18:0/12:0, GalCer d18:1/12:0, GluCer d18:1/12:0, LacCer d18:1/12:0, D7-cholesterol, CE 17:0, MG 17:0, 4ME 16:0 diether DG, D5-TG 16:0/18:0/16:0 (Avanti Polar Lipids, Alabaster, AL). Lipid levels for each sample were calculated by summing up the total number of moles of all lipid species measured by all three LC-MS methodologies and then normalizing the total to mol %. The final data are presented as mean mol % lipid content with error bars representing standard deviation (SD). Of note, a study pool QC was not performed because the analyzed samples were fractions from different organelles, and all the samples from the same organelle were analyzed in a single batch. Instead, a reference plasma QC sample was run across all the batches to assess the performance, revealing a mean intra-batch variability of <10% and inter-batch variability of <20%. RF classification was performed as previously described^13,16^. Briefly, RF (no. of trees = 5000), a machine learning approach, was used to select the best-performing lipid species per pairwise comparison. Selection was based on the lowest mean minimum depth values in the trees (indicating earlier involvement in decision-making, with lower values being better) and the highest frequencies of occurrence in the trees (indicating higher importance). Lipid species with higher frequencies at lower nodes were particularly effective in classifying the different groups^94–96^.

### Statistical analysis

Data are presented as the mean ± SD with all averages based on at least three independent experiments. iPSC-derived neurons from the same individual were differentiated at different times from different NPC passages. Data distributions were confirmed to be normal, and differences among means were assessed by either one-way or two-way analysis of variance (ANOVA), followed by a Dunnett’s or Tukey’s multiple comparison post-hoc test as stated in the figure legend. Statistical analyses were performed using GraphPad Prism (Version 10, GraphPad Software Inc., USA). The null hypothesis was rejected at a level of 0.05. Investigators were not blinded during the quantification of imaging experiments.

### Study approval

An exempt protocol was approved by the Institutional Review Board at Columbia University Irving Medical School for de-identified human pathologic specimens.

## Supplementary information

Supplementary material

## Data Availability

All study data are included in the article and/or SI Appendix.
