# Supplementary material for: The role of alpha-synuclein in synucleinopathy: Impact on lipid regulation at mitochondria–ER membranes
Source: NPJ Parkinsons Dis. 2025 Apr 30;11:103. doi: 10.1038/s41531-025-00960-x (PMC12043847; doi:10.1038/s41531-025-00960-x)
Supplement: Supplementary file 1 — Supplementary material [file 41531_2025_960_MOESM1_ESM.pdf]

Supplementary Table 1. Substantia nigra sample donors' demographic Information				
**Group**	**Sample size**	**Age (Year ± SD)**	**Sex (M/F)**	**PMI (hour ± SD)**
Control	14	80.2 ± 12.3	8/6	14.1 ± 7.5
PD	16	79.7 ± 7.1	12/4	18.6 ± 12.1

Abbreviations: PD, Parkinson's disease; PMI, post-mortem interval; SD, standard deviation; M, male; F, female.

Supplementary Table 2: Lipids detected (>500 species belonging to 31 classes).

Abbr.	Lipid (detectable species)	Abbr.	Lipid (detectable species)
**FC**	Free Cholesterol	**PC**	Phosphatylcholine (25 species)
**CE**	Cholesterol Ester (20 species)	**PCe**	Ether phosphatidylcholine (25 species)
**AC**	Acyl Carnitine (9 species)	**PE**	Phosphatidylethanolamine (25 species)
**MG**	Monoacylglycerol (18 species)	**PEp**	Plasmalogen phosphatidylethanolamine (25 species)
**DG**	Diacylglycerol (28 species)	**PS**	Phosphatidylserine (25 species)
**TG**	Triacylglycerol (42 species)	**PI**	Phosphatidylinositol (25 species)
**dhCer**	Dihydroceramide (12 species)	**PG**	Phosphatidylglycerol (25 species)
**Cer**	Ceramide (12 species)	**BMP**	Bis(monoacylglycero)phosphate (25 species)
**SM**	Sphingomyelin (12 species)	**AcylPG**	Acyl Phosphatidylglycerol (15 species)
**dhSM**	Dihydrosphingomyelin (12 species)	**LPC**	Lysophosphatidylcholine (9 species)
**Sulf**	Sulfatide (18 species)	**LPCe**	Ether lysophosphatidylcholine (9 species)
**MHCer**	Monohexosylceramide (24 species)	**LPE**	Lysophosphatidylethanolamine (9 species)
**LacCer**	Lactosylceramide (24 species)	**LPEp**	Plasmogen Lysophosphatidylethanolamine (9 species)
**GM3**	Monosialodihexosylganglioside (18 species)	**LPI**	Lysophosphatidylinositol (9 species)
**GB3**	Globotriaosylceramide (12 species)	**LPS**	Lysophosphatidylserine (11 species)
**PA**	Phosphatidic acid (25 species)		

Abbreviations: Abbr., abbreviation.

Supplementary Table 3. Striatum sample donors' demographic Information				
**Group**	**Sample size**	**Age (Year ± SD)**	**Sex (M/F)**	**PMI (hour ± SD)**
Control	16	68.2 ± 7.9	13/3	13.5 ± 7.9
PD/DLB	11	77.9 ± 5.9	9/2	16.2 ± 10.9
MSA	10	68.6 ± 8.5	5/5	15.9 ± 9.7

Abbreviations: PD, Parkinson's disease; DLB, dementia with Lewy bodies; MSA, multiple system atrophy; PMI, post-mortem interval; SD, standard deviation; M, male; F, female.

Supplementary Table 4: List of cell lines

Cell name	Status	Sex (M/F)	Age at Biopsy	iPSC reprogramming method	Mutation	Referen ce
KO SNCA	Control with no αSyn	M	67	-	Deletion in Exon 2 leads to loss of αSyn	(1, 2)
Control	Control	M	72	Sendai	-	(3)
Control-2	Control	F	72	Sendai	-	(4)
Control-3	Control	M	67	Lentivirus		(5, 6)
Control-4	Control	F		Retrovirus		(7)
Duplication	PD	F	67	Sendai	SNCA gene locus duplication	(8)

Abbreviations: iPSC, induced pluripotent stem cell; M, male; F, female; KO, knock out; Dup., duplication; PD, Parkinson's disease; αSyn, alpha-synuclein.

Supplementary References:

1. P. Barbuti, *et al.*, Using High-Content Screening to Generate Single-Cell Gene-Corrected Patient-Derived iPS Clones Reveals Excess Alpha-Synuclein with Familial Parkinson's Disease Point Mutation A30P. *Cells* **9** (2020).

2. L. Mahoney-Sanchez, *et al.*, Alpha synuclein determines ferroptosis sensitivity in dopaminergic neurons via modulation of ether-phospholipid membrane composition. *Cell Reports* **40**, 111231 (2022).

3. Z. Hanss, *et al.*, Mitochondrial and Clearance Impairment in p.D620N VPS35 Patient-Derived Neurons. *Movement Disorders* **36**, 704–715 (2021).

4. C. Berenguer-Escuder, *et al.*, Impaired mitochondrial–endoplasmic reticulum interaction and mitophagy in Miro1-mutant neurons in Parkinson's disease. *Human Molecular Genetics* **29**, 1353–1364 (2020).

5. P. A. Barbuti, *et al.*, Generation of two iPS cell lines (HIHDNDi001-A and HIHDNDi001-B) from a Parkinson's disease patient carrying the heterozygous p.A30P mutation in SNCA. *Stem Cell Research* **48C**, 101951–101951 (2020).

6. D. C. Schöndorf, *et al.*, IPSC-derived neurons from GBA1-associated Parkinson's disease patients show autophagic defects and impaired calcium homeostasis. *Nature Communications* **5**, 4028–4028 (2014).

7. M. J. Devine, *et al.*, Parkinson's disease induced pluripotent stem cells with triplication of the alpha-synuclein locus. *Nature Communications* **2**, 410–440 (2011).

8. K. Ishikawa, *et al.*, Generation of three clones (JUCGRMi002-A, B, C) of induced pluripotent stem cells from a Parkinson's disease patient with *SNCA* duplication. *Stem Cell Research* **74**, 103296 (2024).

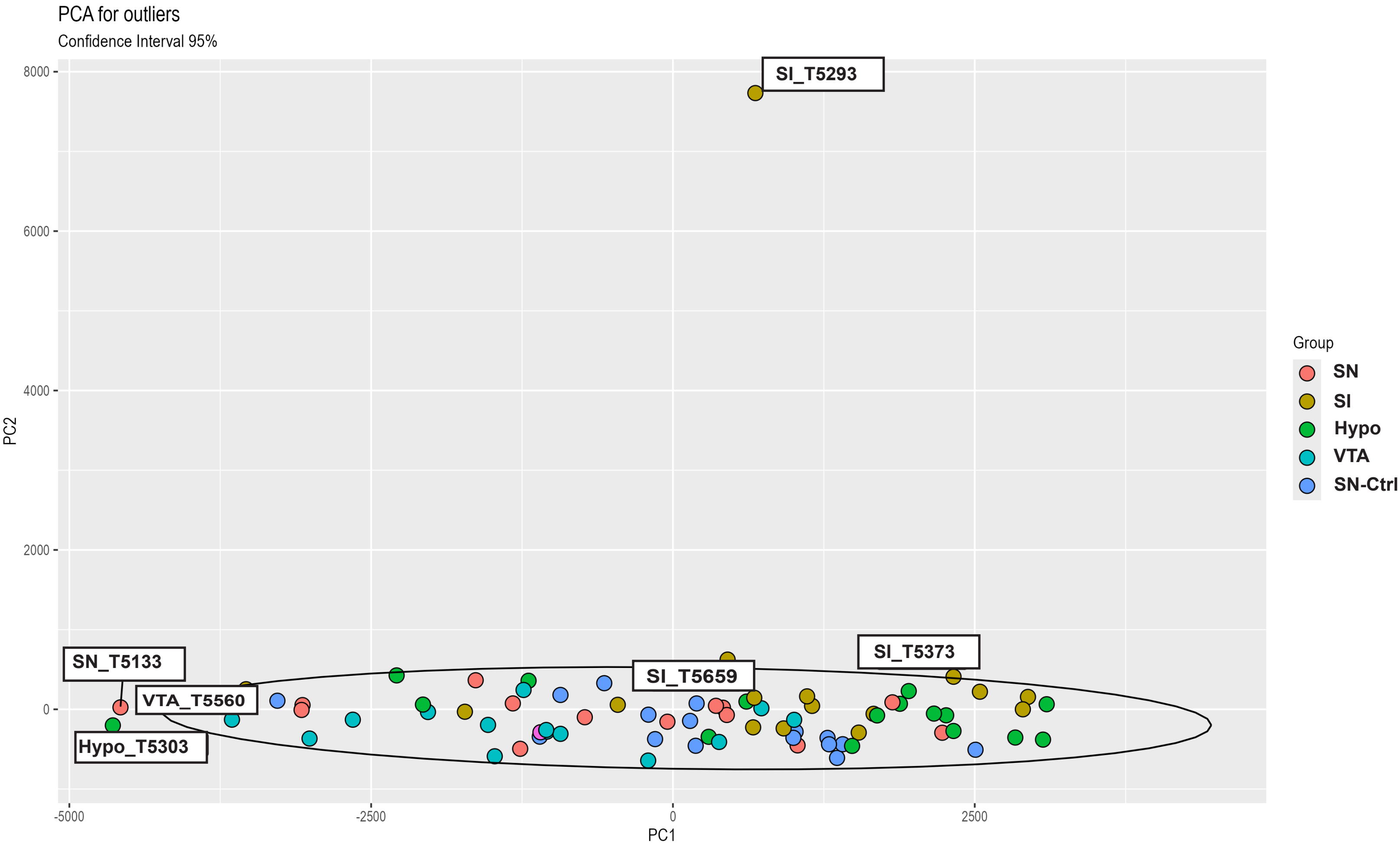

Supplementary Fig.1. Principle Component Analysis (PCA) of postmortem brain regions. Sam-ples outside the 95% confidence interval (red eliptical box) were excluded from this study. Abbrevia-tions: substantia nigra (SN), substantia innominata (SI), hypothalamus (Hypo), ventral tegmental area (VTA), SN from non PD donor controls (SN-Ctrl).

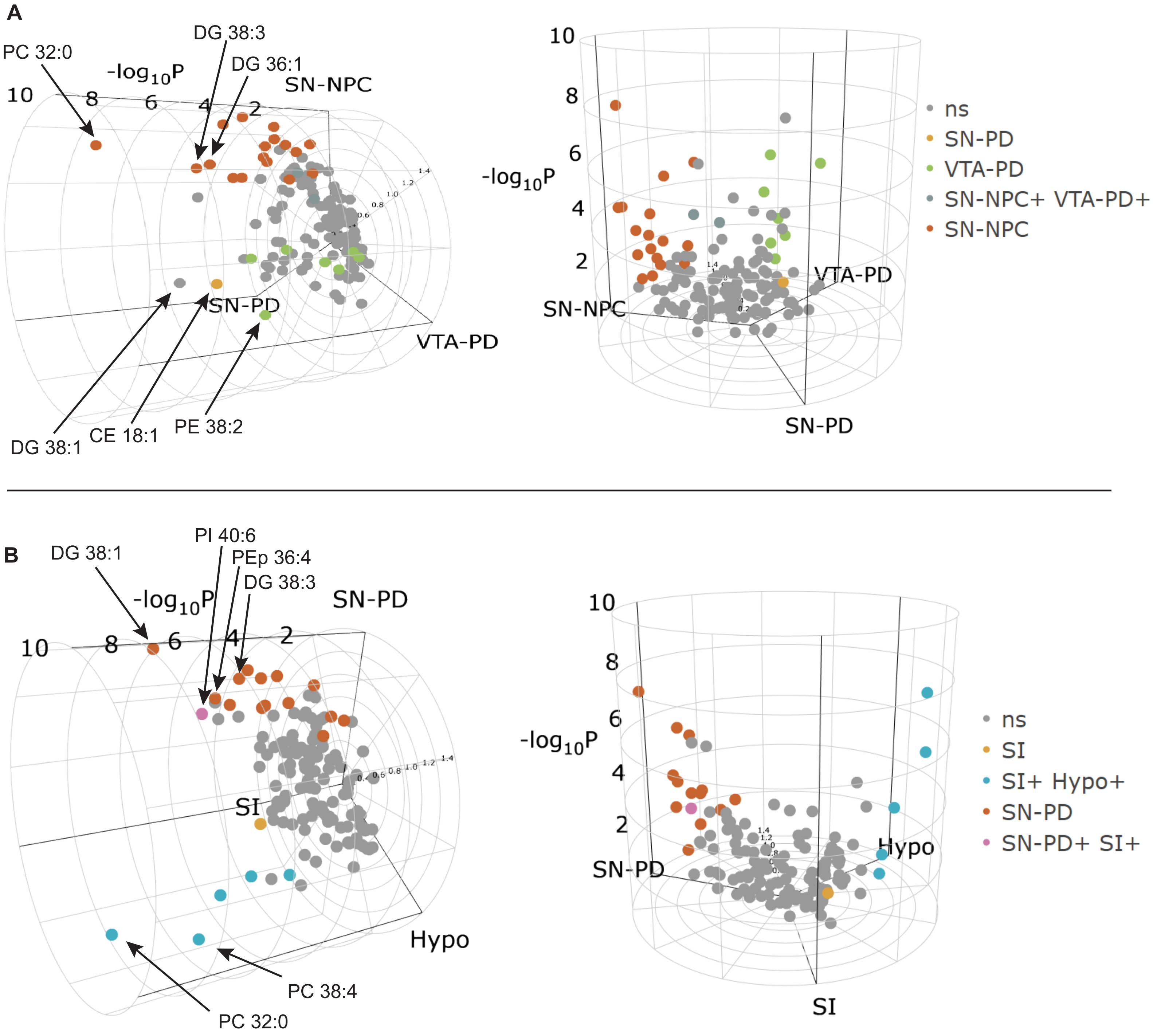

Supplementary Fig.2A. Cylindrical volcano plots of significantly altered lipids from different brain regions. 3D volcano plots were assembled using the p values calculated from a one-way ANOVA, followed by pairwise t test corrected by false discovery rate (FDR) of 5 tissues: substantia nigra (SN), ventral tegmental area (VTA), hypothalamus (Hypo), substantia innominata (SI), all from PD donors, and the substantia nigra from non PD control donors (NPC). The Z axis of the volcano plot shows ANOVA p values (-log10) for the associated lipid species for the subset of 3 groups as illustrated in: **(A)** SN-PD, VTA-PD, SN-NPC; and **(B)** SN-PD, SI, Hypo. The radial axis corresponds to the Z-score. The data for 3 subset groups were reduced to a 2D polar coordinate system. Colored dots indicate which species are altered significantly by adjusted p values in pairwise comparisons.

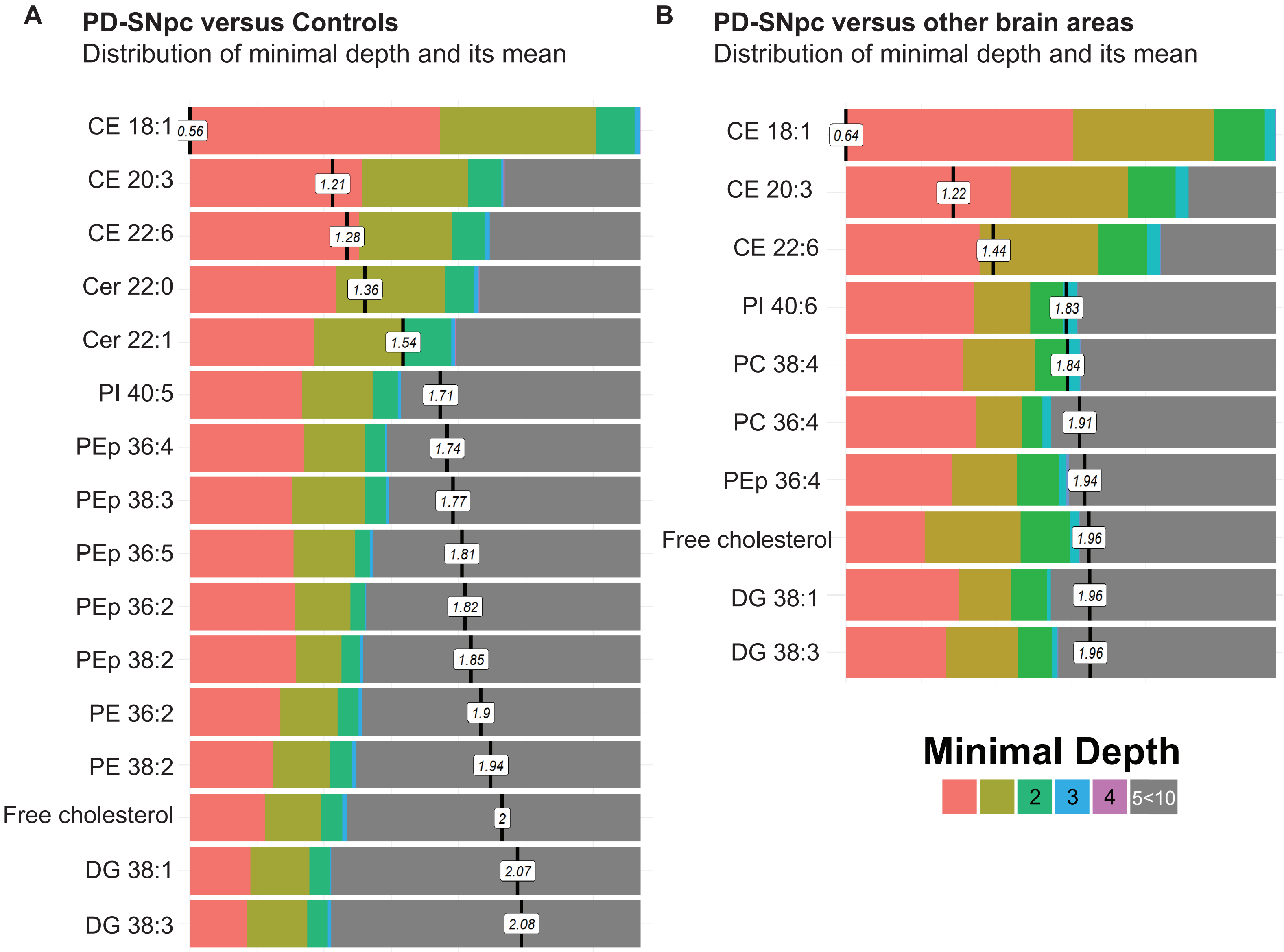

Supplementary Fig.2B. Variable importance based on minimum depth from Random Forest (RF) analysis. (A) PD vs Controls at the SNpc (B) PD-SNpc vs PD-other brain areas. Note, minimal depth indicates how early a lipid is involved in decision trees. Higher frequencies at lower nodes indicate that specific lipid species effectively classify the different groups.

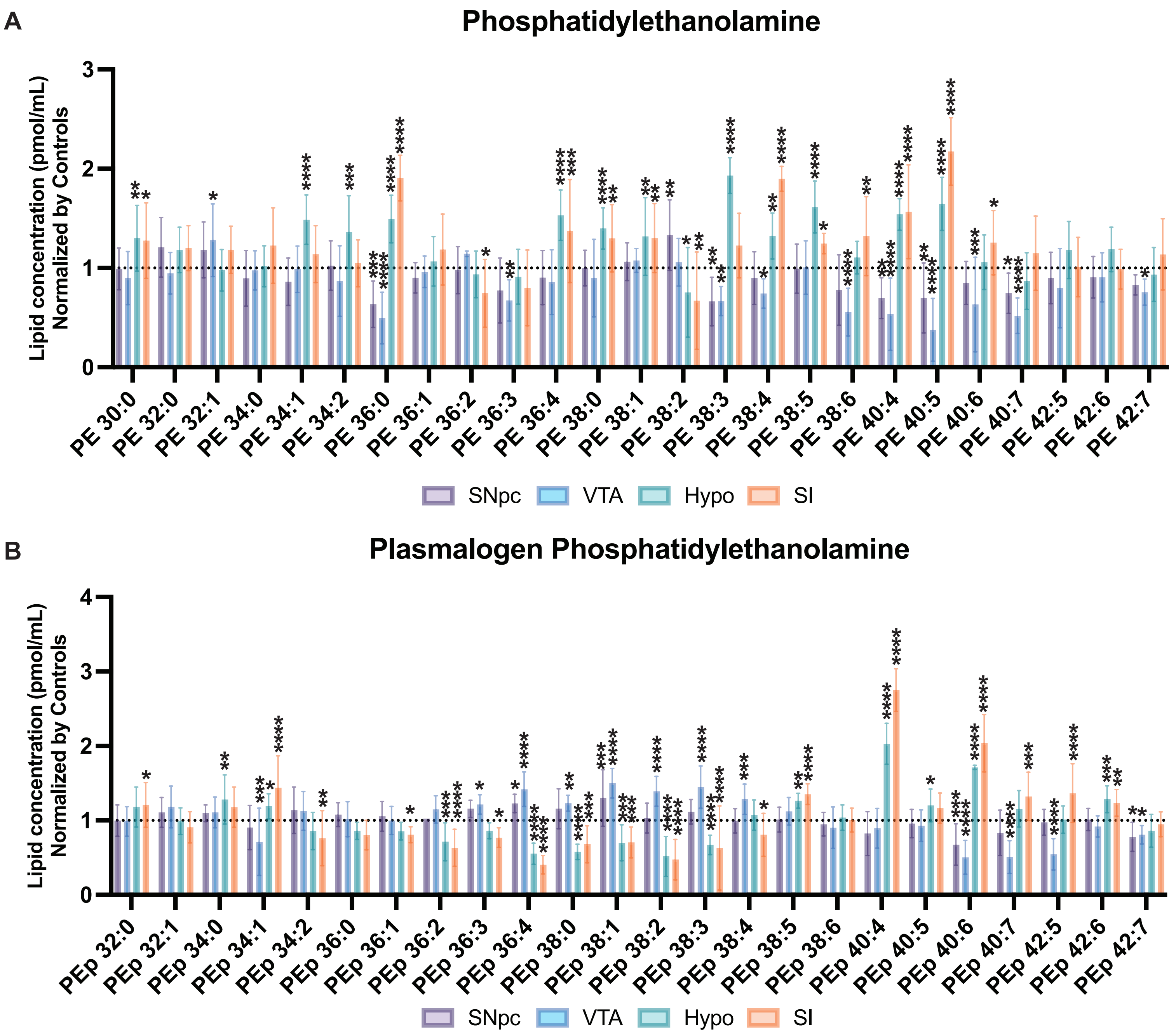

Supplementary Figure 2C: Lipid alterations in the postmortem Parkinson's disease (PD) brain. Relative concentrations of the indicate lipid species of: (**A**) Phosphatidylethanolamine (PE) and (**B**) Plasmalogen Phosphatidylethanolamine (PEp) in homogenates of different brain regions: substantia nigra pars compacta (SNpc), ventral tegmental area (VTA), hypothalamus (Hypo), substantia innominata (SI) of PD postmortem donors (n=16 for each brain region). Lipid species were normalized to concentrations in Control samples (n=14) (indicated by dotted lines). Data is presented as the mean ± SD of all independent biological replicates (n) analyzed by an ordinary two-way ANOVA versus the means of the Control group. (A) PE: Interaction ($F_{(96,1825)}$ = 14.02; ****$p<0.0001$), (B) PEp: Interaction ($F_{(92,1752)}$ = 36.47; ****$p<0.0001$). For post-hoc analysis a Dunnett's multiple comparison test, with a single pooled variance was used. *$p < 0.05$, ** $p < 0.01$, ***$p < 0.001$ ****$p < 0.0001$.

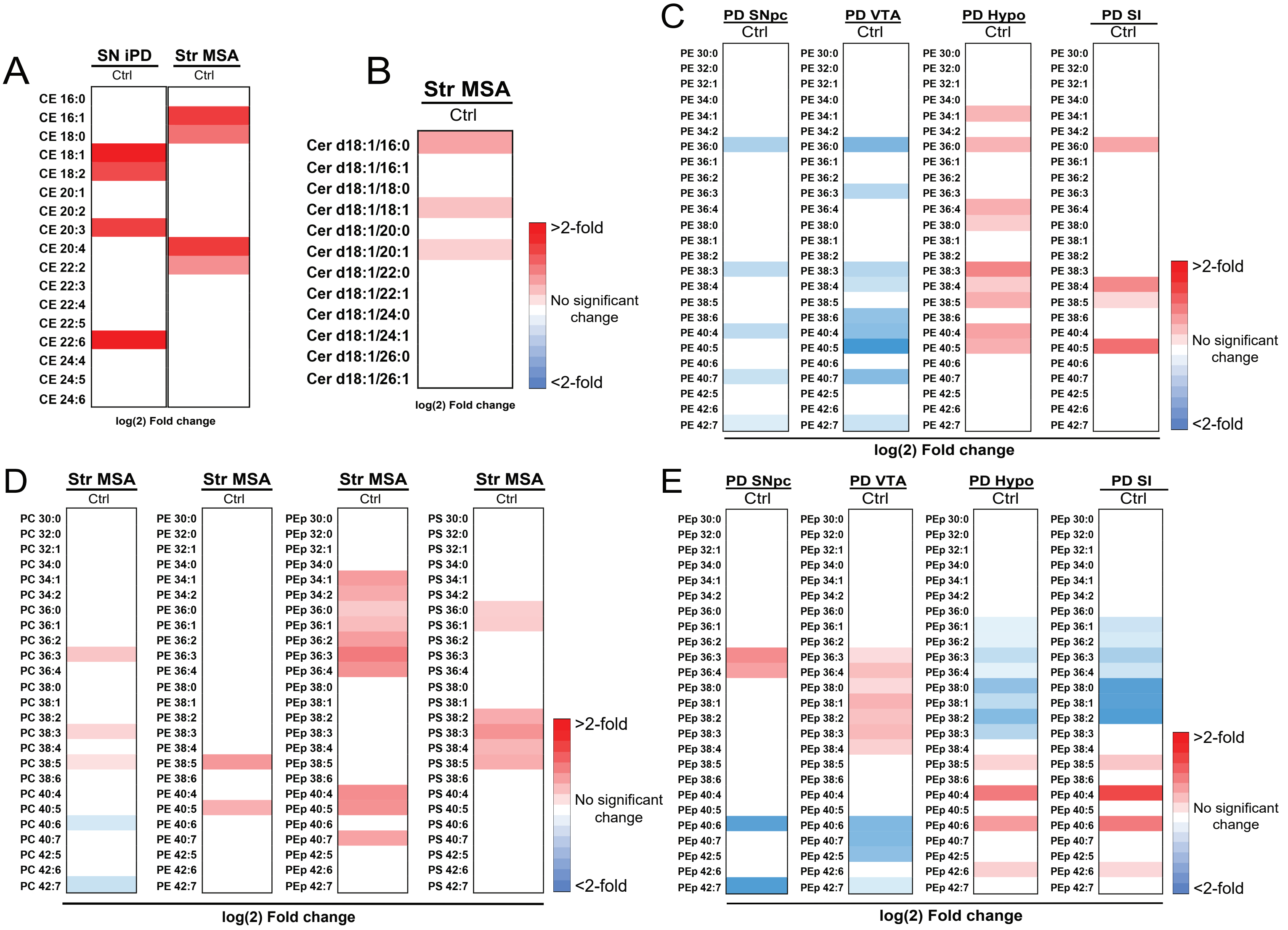

Supplementary Fig.3A. Lipid alterations in postmortem brains. Lipidomic heat map showing Log(2) fold changes in different brain regions of postmortem Parkinson's disease (PD) and multiple system atrophy (MSA) donors. **(A)** Fold-increases in the levels of specific cholesterol esters (CE) in the substantia nigra pars compacta (SN) of PD donors and striatum (Str) of MSA donors compared to Controls. **(B)** Increases in the levels of ceramide (Cer) species in the striatum of MSA donors compared to Controls. **(C)** Alterations of phosphatidylethanolamine (PE) lipid species in different regions of PD brain (substantia nigra pars compacta (SNpc), ventral tegmental area (VTA), hypothalamus (Hypo), substantia innominata (SI)) at postmortem. **(D)** Alterations in different species of phospholipids (phosphatidylcholine (PC), PE, plasmalogen phosphatidylthanolamine (PEp) and phosphatidylserine (PS)) in the striatum of MSA donors compared to controls. **(E)** Alterations in different species of PEp in different regions of PD brains at postmortem compared to controls.

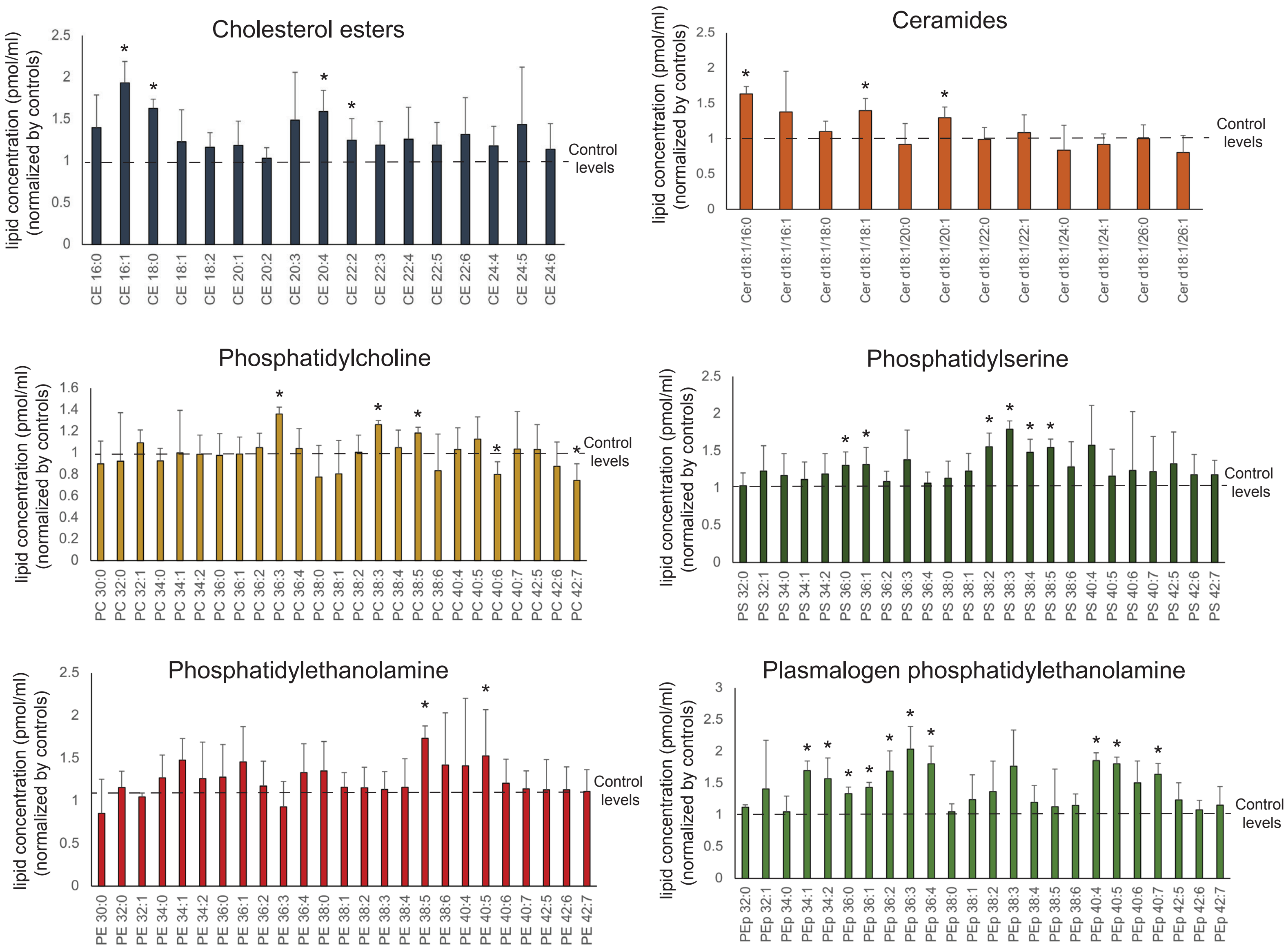

Supplementary Figure 3B: Lipid alterations in post-mortem MSA brains at the straitum. Alterations in individual lipid species of cholesterol esters, ceramides, phosphatidylcholine, phosphatidylserine, phosphatidylethanolamine and plasmalogen phosphatidylethanolamine versus non-MSA, non-PD straitum controls.

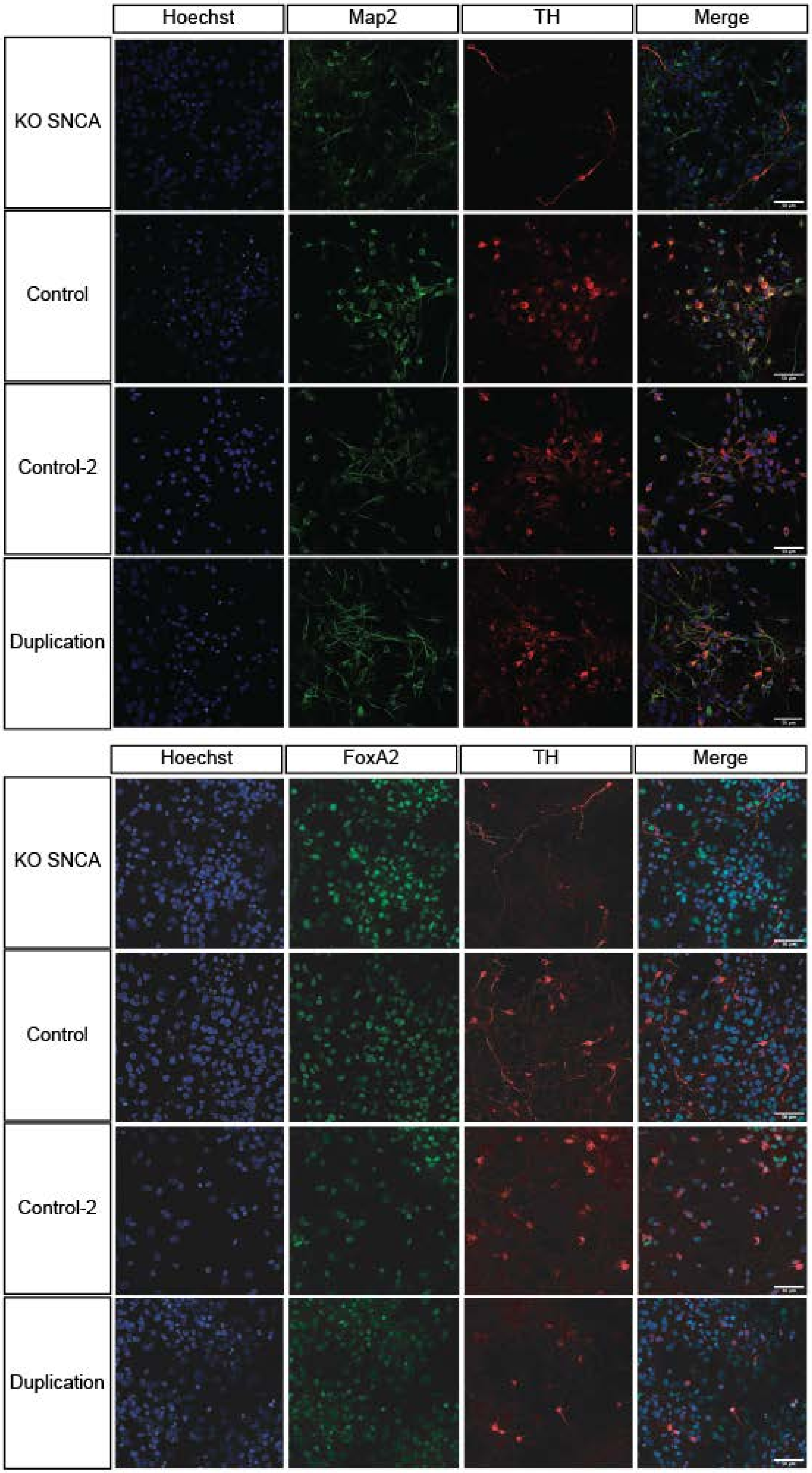

Supplementary Fig.4A. Characterization of midbrain dopaminergic neurons. Representative images of neurons fluorescently labelled with the mature neuronal marker Map2, the rate-limiting enzyme for catecholamine biosynthesis and the biosynthesis of dopamine tyrosine hydroxylase (TH), and the ventral midbrain marker FoxA2.

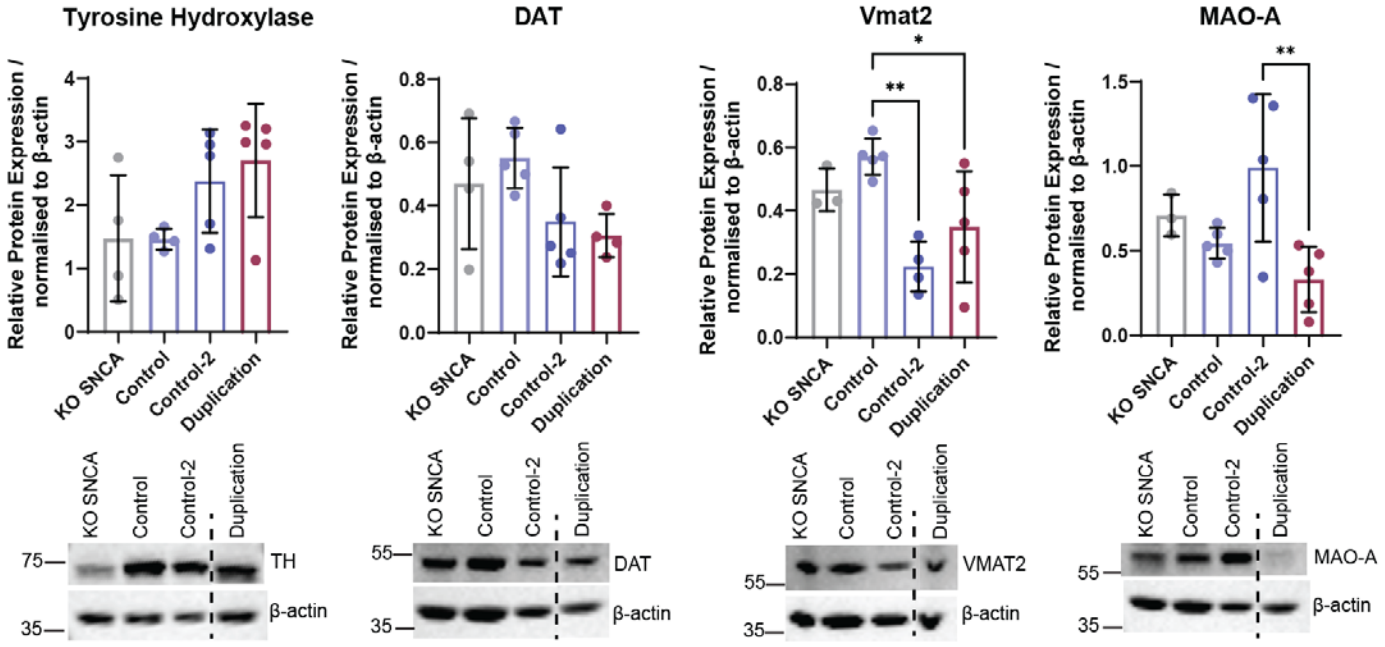

Supplementary Fig.4B. Neuronal chacterization. Cell lines were positive for markers of dopaminergic neurons as shown by a representative western blot. Data are means ± SD of a minimum of 3 independent biological replicates (n) analyzed by an ordinary one-way ANOVA, for statistical analyses of the indicated proteins a Tukey's multiple comparison post-hoc was used with a single pooled variance. Tyrosine Hydroxylase (TH) (Interaction $F_{3,14}$ =2.821; p=0.0772), DAT (Interaction $F_{3,14}$ =2.706; p=0.0852), VMAT2 (Interaction $F_{3,13}$ =7.841; **p=0.0031), MAO-A (Interaction $F_{3,14}$ =5.522; *p=0.0103). In all statistical analyses: *p<0.05, **p<0.01, ***p<0.001, ****p<0.0001.

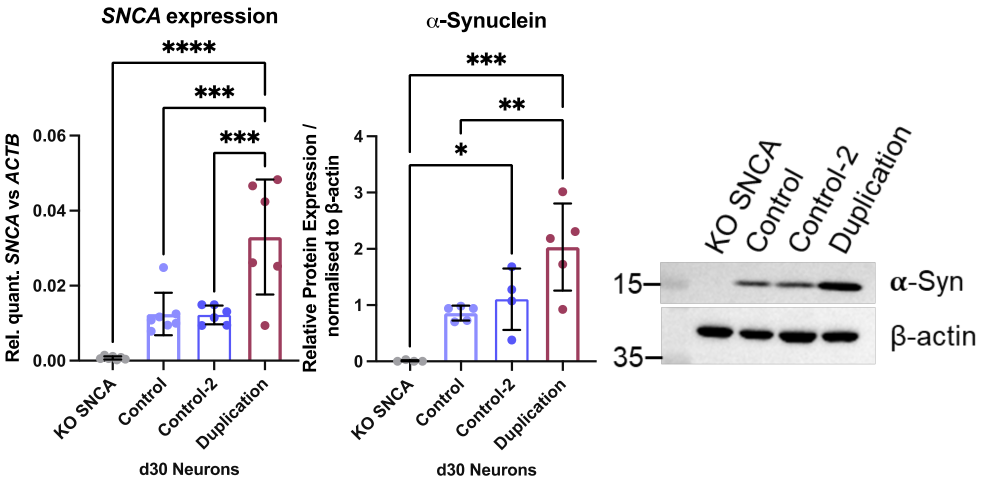

Supplementary Fig. 4D. Validation of iPS-derived neurons expressing SNCA. Gene and protein expression of αSyn after 30-days of directed neuronal differentiation. For SNCA gene expression the data are means ± SD of a minimum of 6 independent biological replicates (n) analyzed by an ordinary one-way ANOVA with (A) Interaction $F_{3,22}$ =17.98; ****p<0.0001. Characterization of αSyn protein level by western blot, the data was obtained using the means ± SD of a minimum of 4 independent biological replicates (n) analyzed by an ordinary one-way ANOVA (Interaction $F_{3,14}$ =13.04; ***p=0.0002) with a representative image shown. For all statistical analysis, a Tukey's multiple comparison post-hoc was used with a single pooled variance.

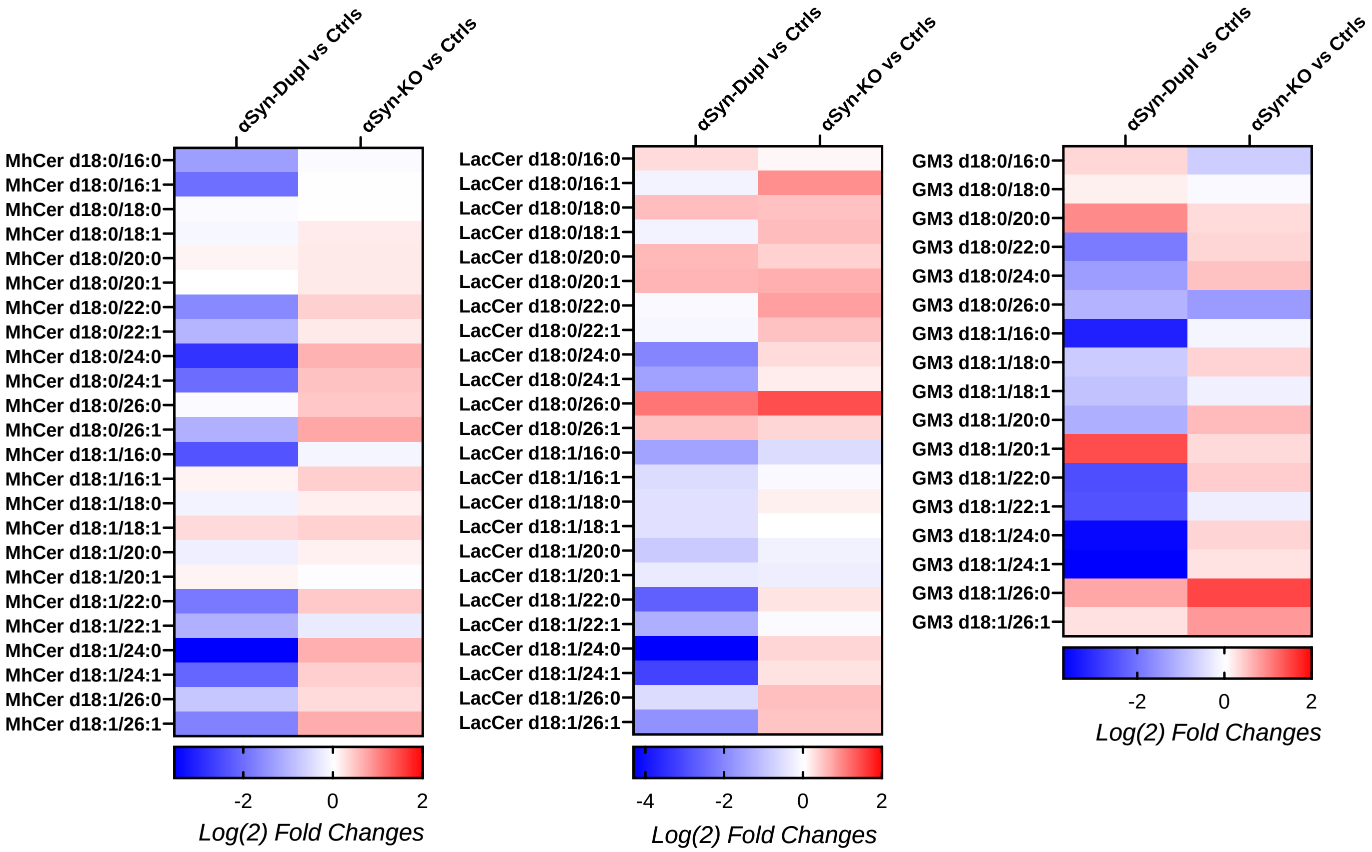

Supplementary Fig.4E. Altered complex sphingolipid and glycolipid classes in patient-derived neurons expressing differing αSyn dosage. Lipidomic heat map of Log(2) fold changes of selected lipid species in iPS-derived neurons carrying altered αSyn dosage compared to Controls. Lipid abbreviations: MhCer: Monohexosylceramide; LacCer: Lactosylceramide; GM3: Monosialodihexosylganglioside.

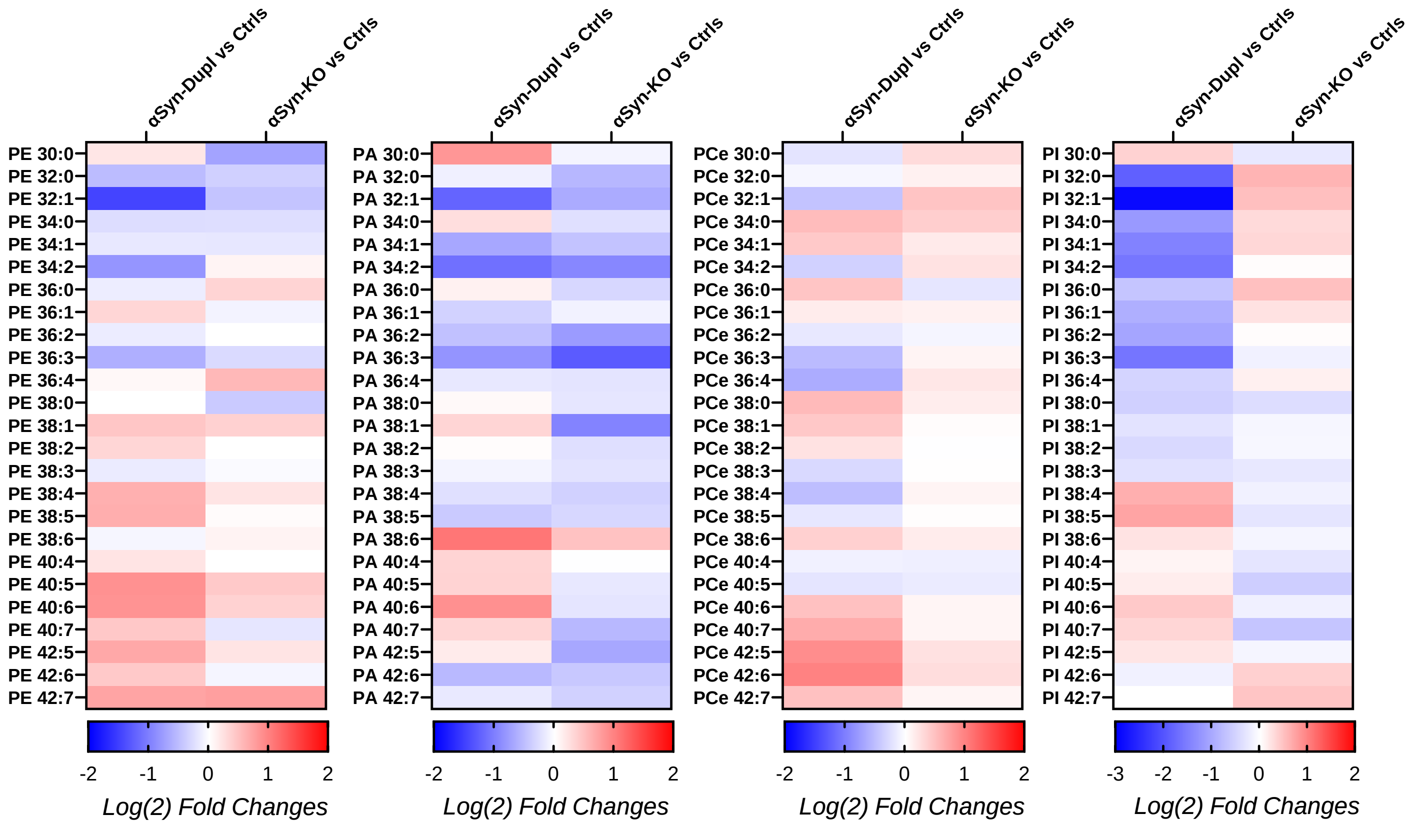

Supplementary Fig. 4F: Altered phospholipid classes in neurons expressing differing αSyn dosage. Lipidomic heat map of Log(2) fold changes of selected lipid species in iPS-derived neurons carrying altered αSyn dosage compared to Controls. Lipid abbreviations: PE: Phosphatidylethanolamine; PA: Phosphatidic Acid; PCe: Ether phosphatidylcholine; PI: Phosphatidylinositol.

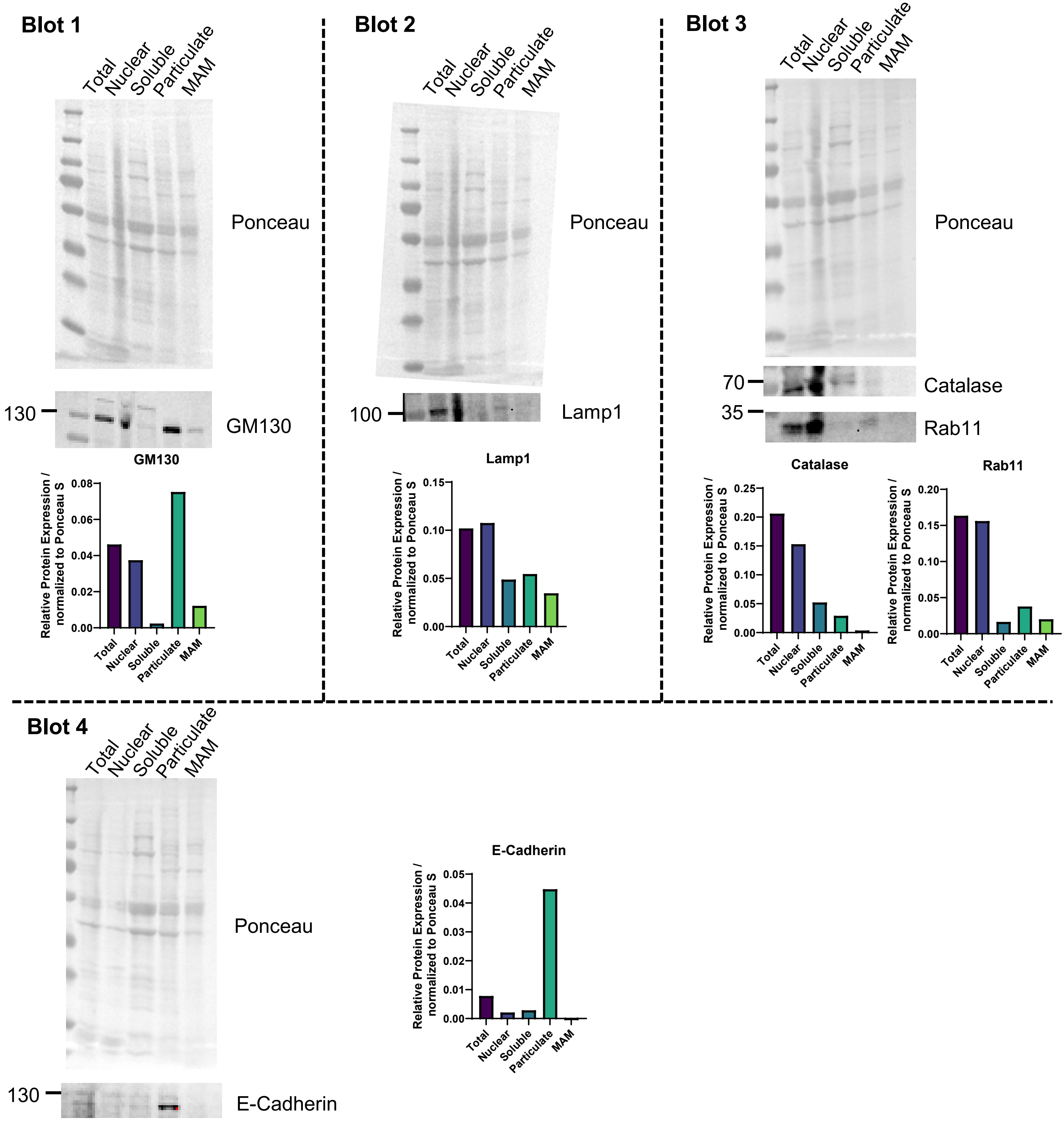

Supplementary Figure 4G: Validation of MAM purity. To assess the relative purity of the MAM fraction, neurons were lysed after 30 days of directed differentiaton to either isolate the MAM fraction as previously described[1,2], or a subcellular fraction of membrane particulates[3]. Neurons fractions were probed for a panel of organelle markers (Golgi Body - GM130; Lysosome - Lamp1; Peroxisome - Catalase; Endosome - Rab11; Plasma Membrane - E-Cadherin) against total protein level (Ponseau S). Abbreviations: Total - total homogenate; Nuclear - nuclear enriched subcellular fraction; Soluble - soluble post-nuclear fraction; Particulate - particulate membrane fraction.

References

1. Area-Gomez, E. Assessing the Function of Mitochondria-Associated ER Membranes. Methods in Enzymology 547, 181–197 (2014).
2. Montesinos, J. & Area-Gomez, E. Isolation of mitochondria-associated ER membranes. in Methods in Cell Biology (eds. Pon, L. A. & Schon, E. A.) vol. 155 33–44 (Academic Press, Cambridge, MA, USA, 2020).
3. Kikuchi, H. et al. Spinal cord endoplasmic reticulum stress associated with a microsomal accumulation of mutant superoxide dismutase-1 in an ALS model. Proceedings of the National Academy of Sciences 103, 6025–6030 (2006)

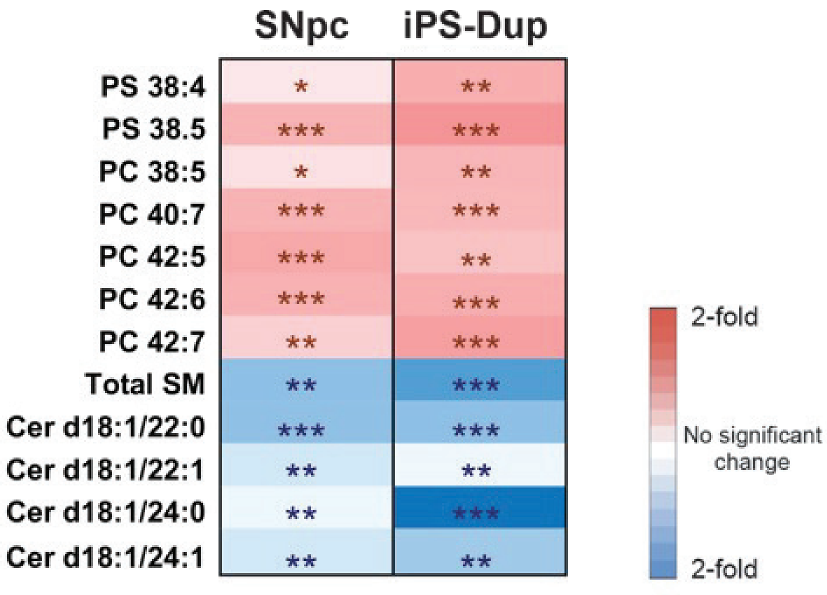

Supplementary Figure 5: Common changes found in IPSC and SNpc tissues. Heatmap shows the degree and directionality of change in the concentration of the indicated lipid classes and species in SNpc tissues and iPSCs compared to their respective controls. Stars within each cell represent the statistical significance of slope of change for each of these lipid species (*p < 0.05, **p < 0.01, ***p < 0.001).

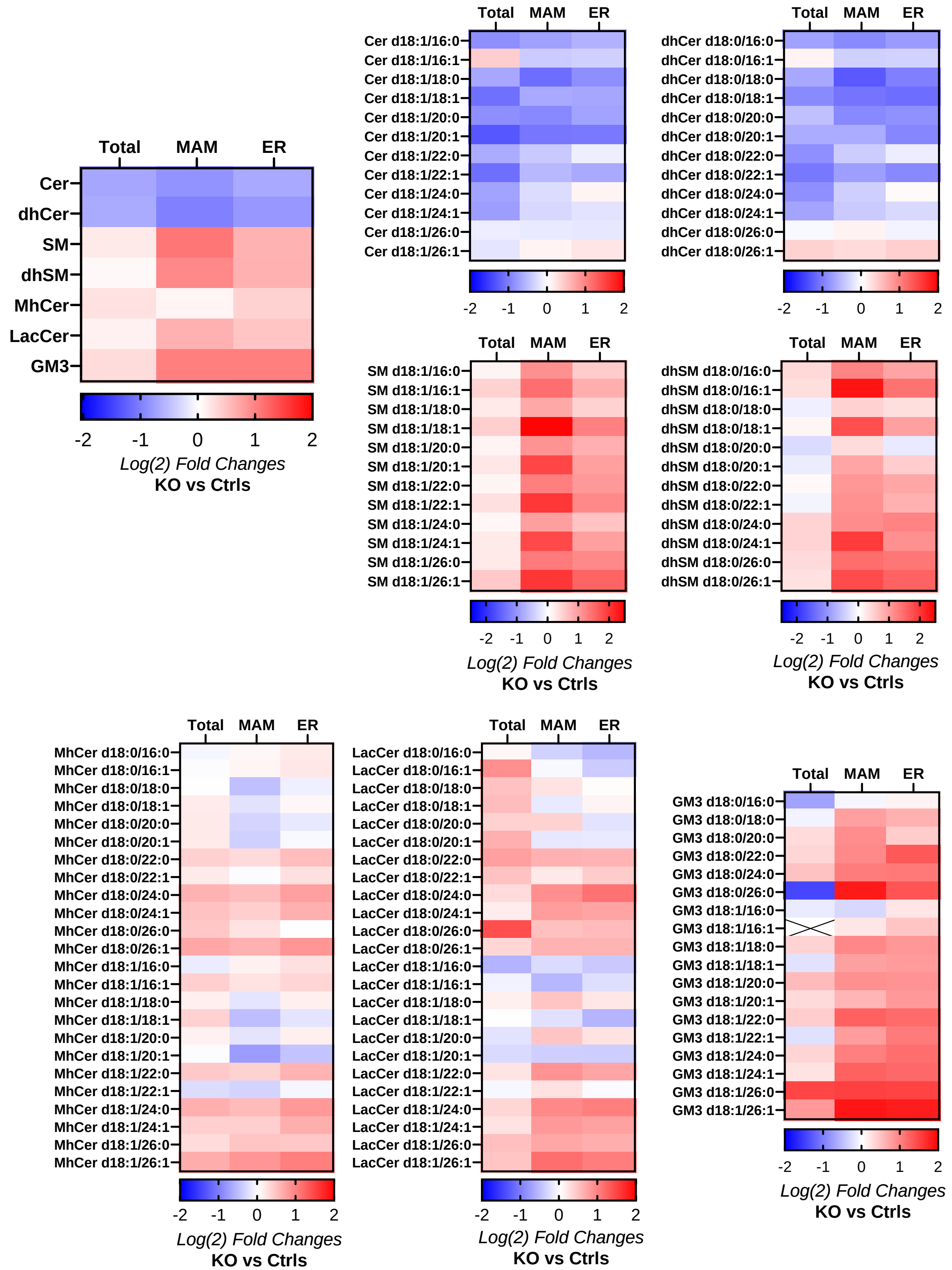

Supplementary Fig.6A. Lipid alterations in αSyn-KO neurons across different subcellular fractions. Lipidomic heat map of Log(2) fold changes of sphingolipid and glycolipid lipid classes and lipid species in iPS-derived neurons carrying αSyn-KO compared to Controls in the total non-fractionated homogenate, MAM and bulk-ER. Lipid abbreviations: Cer: Ceramide; dhCer: Dihydroceramide; SM: Sphingomyelin; dhSM: Dihydrosphingomyelin; MhCer: Monohexosylceramide; LacCer: Lactosylceramide; GM3: Monosialodihexosylganglioside.

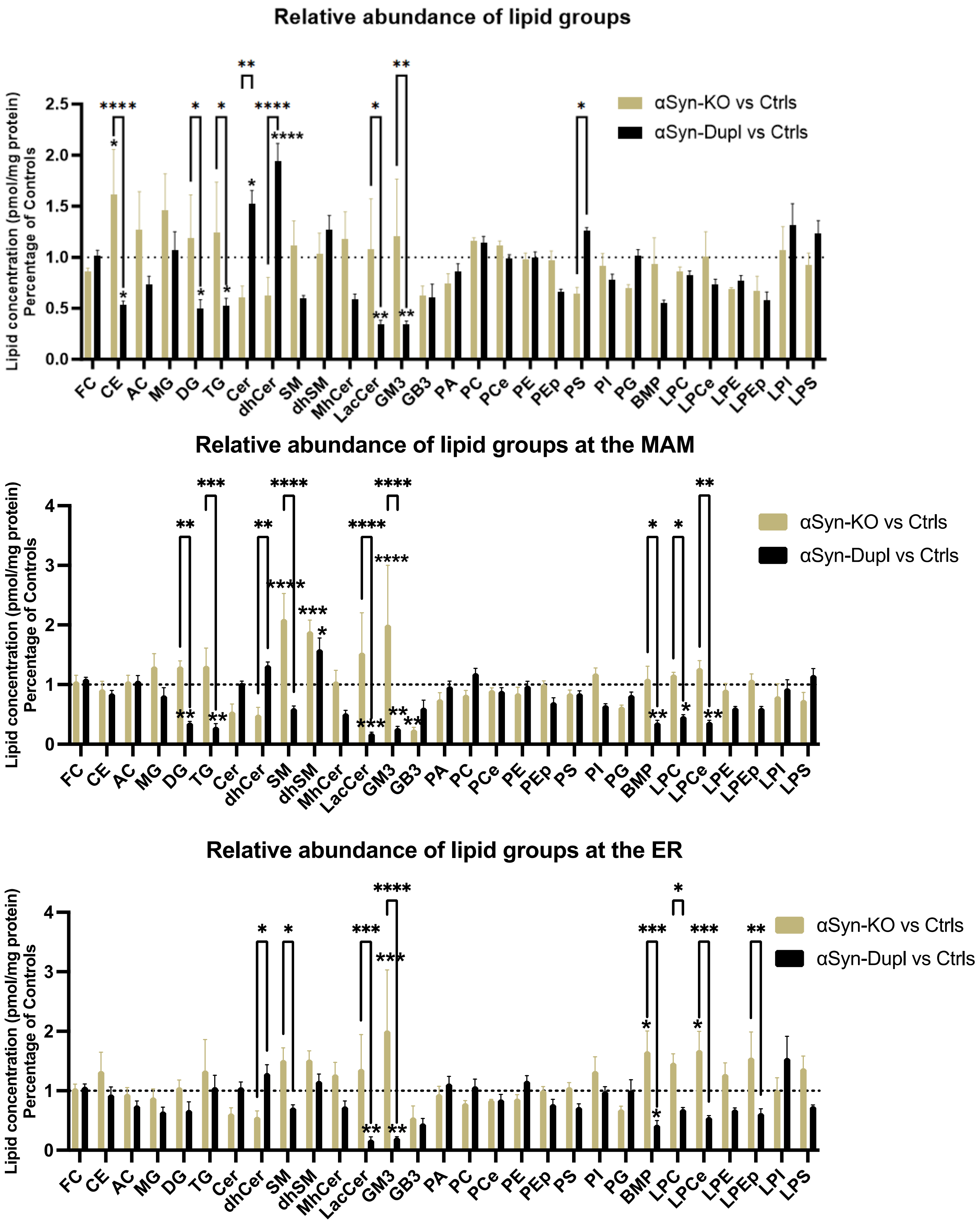
Relative abundance of lipid groups
Lipid concentration (pmol/mg protein) Percentage of Controls
0.0
0.5
1.0
1.5
2.0
2.5
αSyn-KO vs Ctrls
αSyn-Dupl vs Ctrls
FC CE AC MG DG TG Cer dhCer SM dhSM MhCer LacCer GM3 GB3 PA PC PCe PE PEp PS PI PG BMP LPC LPCe LPE LPEp LPI LPS
Relative abundance of lipid groups at the MAM
Lipid concentration (pmol/mg protein) Percentage of Controls
0
1
2
3
4
αSyn-KO vs Ctrls
αSyn-Dupl vs Ctrls
FC CE AC MG DG TG Cer dhCer SM dhSM MhCer LacCer GM3 GB3 PA PC PCe PE PEp PS PI PG BMP LPC LPCe LPE LPEp LPI LPS
Relative abundance of lipid groups at the ER
Lipid concentration (pmol/mg protein) Percentage of Controls
0
1
2
3
4
αSyn-KO vs Ctrls
αSyn-Dupl vs Ctrls
FC CE AC MG DG TG Cer dhCer SM dhSM MhCer LacCer GM3 GB3 PA PC PCe PE PEp PS PI PG BMP LPC LPCe LPE LPEp LPI LPS

Supplementary Fig.6B. Relative abundance of lipid groups expressed in patient-derived neurons differing αSyn dosage in different cellular subcellular compartments. Relative lipid concentration (pmol/mg protein) normalized to controls. Data are means ± SEM of a minimum of 3 independent biological replicates (n) analyzed by an ordinary two-way ANOVA. Post-hoc analysis was performed using Tukey's multiple comparison test, for the relative abundance of lipid groups at the Total homogenate: Interaction ($F_{(56,348)}$ = 2.802; ****$p<0.0001$); the MAM: Interaction ($F_{(56,348)}$ = 3.533; ****$p<0.0001$), and at the ER: Interaction ($F_{(56,348)}$ = 2.257; ****$p<0.0001$). Lipid abbreviations: FC: Free Cholesterol; CE: Cholesterol Ester; AC: Acyl Carnitine; MG: Monoacylglycerol; DG: Diacylglycerol; TG: Triacylglycerol; Cer: Ceramide; dhCer: Dihydroceramide; SM: Sphingomyelin; dhSM: Dihydrosphingomyelin; MhCer: Monohexosylceramide; LacCer: Lactosylceramide; GM3: Monosialodihexosylganglioside; GB3: Globotriaosylceramide; PA: Phosphatidic Acid; PC: Phosphatidylcholine; PCe: Ether phosphatidylcholine; PE: Phosphatylethanolamine;PEp:Plasmalogen phosphatidylethanolamine; PS:Phosphatidylserine PI: Phosphatidylinositol; PG:Phosphatidylglycerol; BMP: Bis(monoacylglycerol)phosphate; LPC: Lysophosphatidylcholine; LPCe: Ether Lysophosphatidylcholine; LPE: Lysophosphatidylethanolamine; LPEp: Plasmalogen Lysophosphatidylethanolamine; LPI: Lysophosphatidylinositol; LPS: Lysophosphatidylserine.

Subcellular fractions from αSyn-KO iPS-derived neurons vs Controls

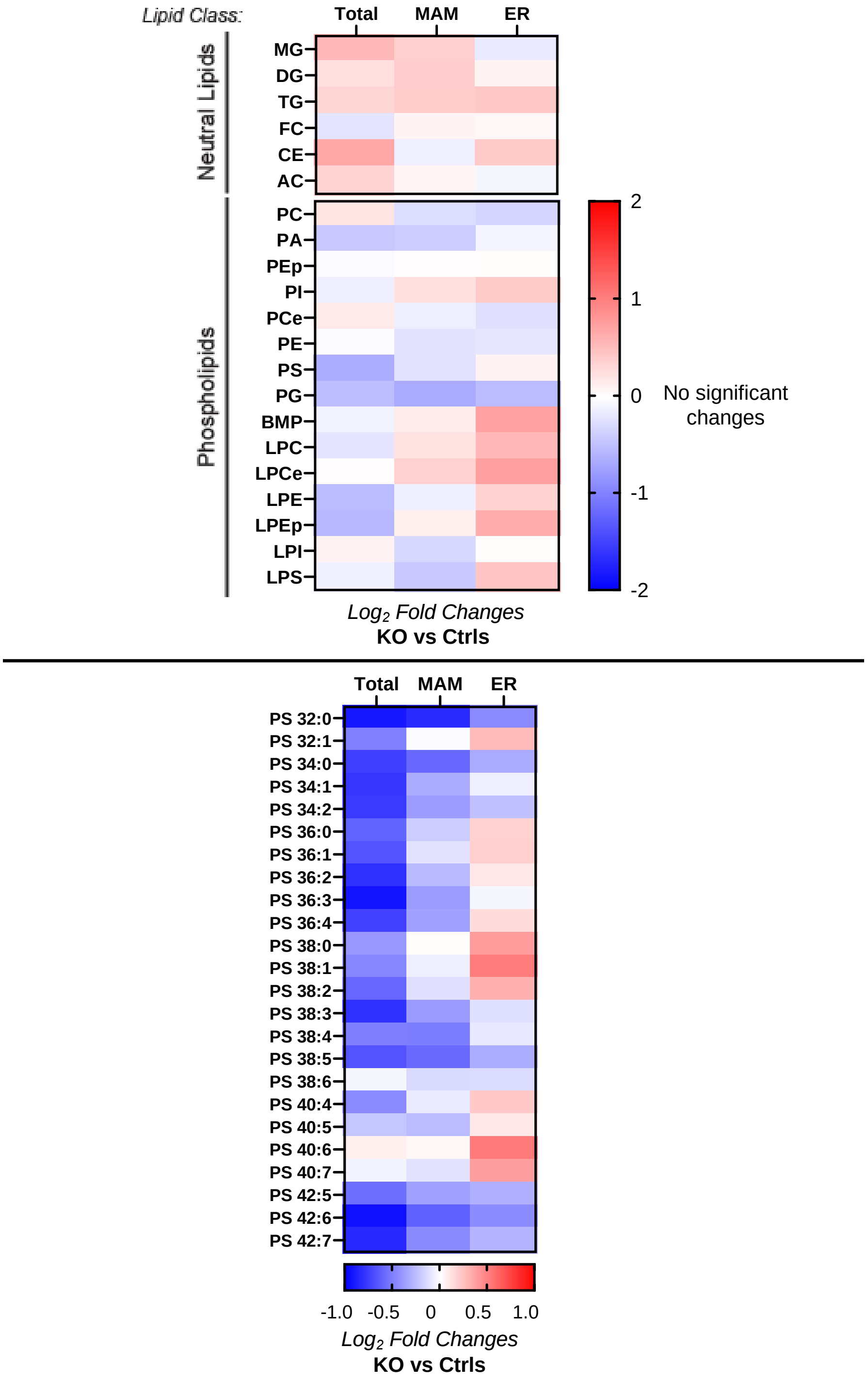

Supplementary Fig.6C. Lipid alterations in αSyn-KO neurons across different subcellular fractions. Lipidomic heat map of Log_2 fold changes of neutral lipid and phospholipid lipid classes and PS lipid species in iPS-derived neurons carrying αSyn-KO compared to Controls in the total non-fractionated homogenate, MAM and bulk-ER. Lipid abbreviations: MG: Monoacylglycerol; DG: Diacylglycerol; TG: Triacylglycerol; FC: Free Cholesterol; CE: Cholesterol Ester; AC: Acyl Carnitine; PC: Phosphatidylcholine; PA: Phosphatidic Acid; PEp: Plasmalogen phosphatidylethanolamine; PI: Phosphatidylinositol; PCe: Ether phosphatidylcholine; PE: Phosphatidylethanolamine;; PS: Phosphatidylserine; PG: Phosphatidylglycerol; BMP: Bis(monoacylglycerol)phosphate; LPC: Lysophosphatidylcholine; LPCe: Ether Lysophosphatidylcholine; LPE: Lysophosphatidylethanolamine; LPEp: Plasmalogen Lysophosphatidylethanolamine; LPI: Lysophosphatidylinositol; LPS: Lysophosphatidylserine

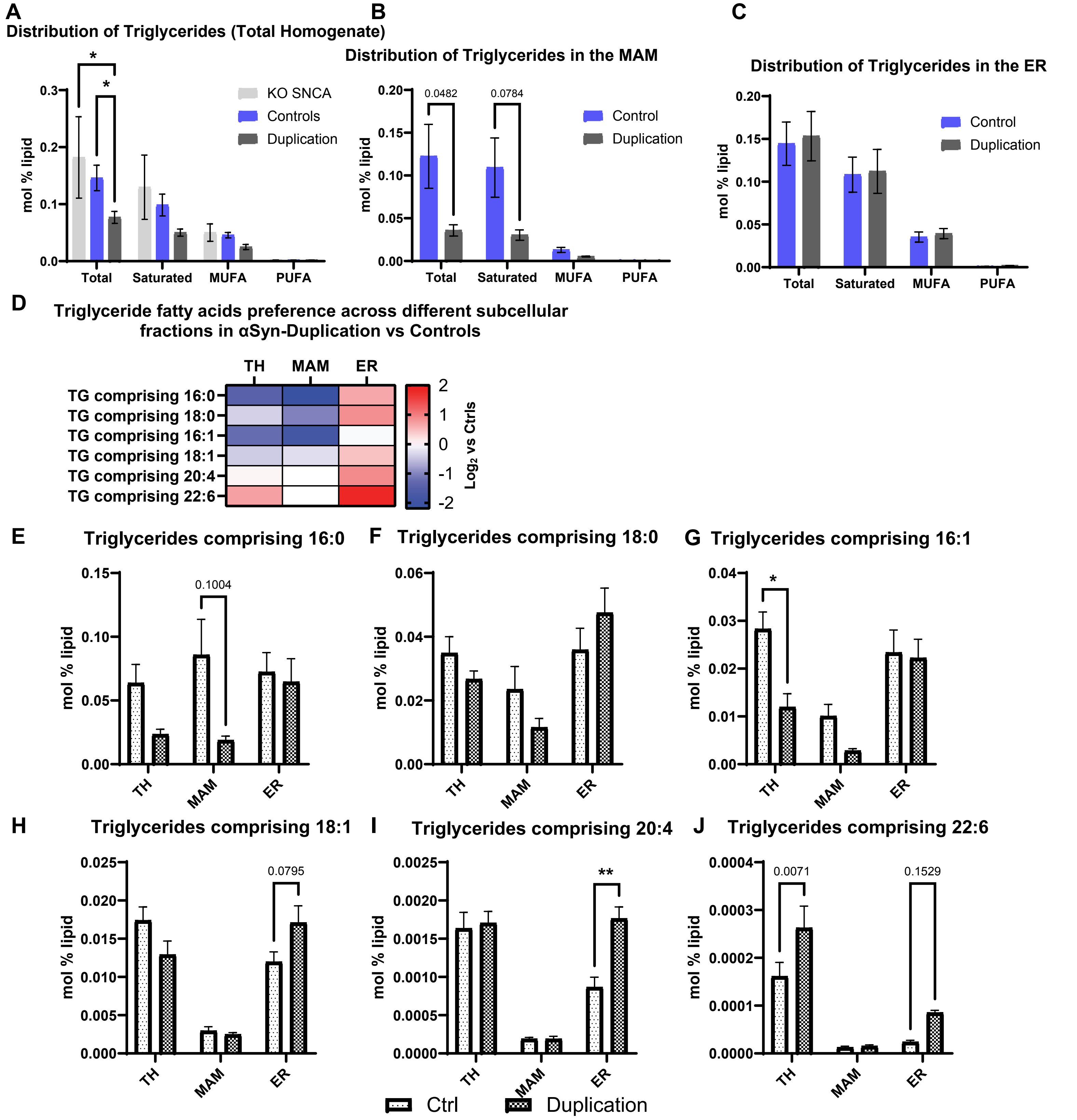

Supplementary Figure 6D: Alpha-synuclein level alters fatty acid preference for triglycerides across subcellular fractions. Distribution of triglycerides based on preference for saturated fatty acids, monounsaturated (MUFA), or polyunsaturated fatty acids (PUFA) in (**A**) total, (**B**) MAM and (**C**) ER subcellular fractions. For statistical analyses (**A-C**) ordinary two-way ANOVAs were perform followed by a Tukey's multiple comparison test with a single pooled variance, giving the following (**A**) Column (Cell line) factor ($F_{(2,48)}$=4.935; *p=0.0112), (**B**) Column (Cell line) factor ($F_{2,48}$=4.580; *p=0.0151), and (**C**) Column (Cell line) factor ($F_{2,48}$= 0.9502; p=0.3938). (**D**) Log_2 fold change difference in the preference of TG comprising different fatty acids (16:0 - Palmitic Acid;18:0 - Stearic Acid; 16:1 - Palmitoleic Acid; 18:1 - Oleic Acid; 20:4 - Arachidonic Acid; 22:6 - Docosahexaenoic Acid); across different subcellular fractions in neurons carrying accumulated αSyn compared to controls carrying normal levels of αSyn. (**E-J**) Quantification of triglycerides comprising different fatty acids in (**D**) across total, MAM and ER subcellular fractions. For statistical analyses (**E-J**) ordinary two-way ANOVAs were perform followed by a Sidak's multiple comparison test with a single pooled variance, giving (E) Column (Cell line) factor ($F_{1,30}$=4.818; *p=0.0360), (**F**) Column (Cell line) factor ($F_{1,30}$=0.2447; p=0.6244), (**G**) $F_{1,30}$= 6.293; *p=0.0178); (**H**) $F_{1,30}$= 0.002001; p=0.9646); (**I**) $F_{1,30}$= 6.145; *p = 0.0190); (**J**) $F_{1,30}$= 9.745; **p=0.0040). In all statistical analyses: *p<0.05, **p<0.01, ***p<0.001, ****p<0.0001.

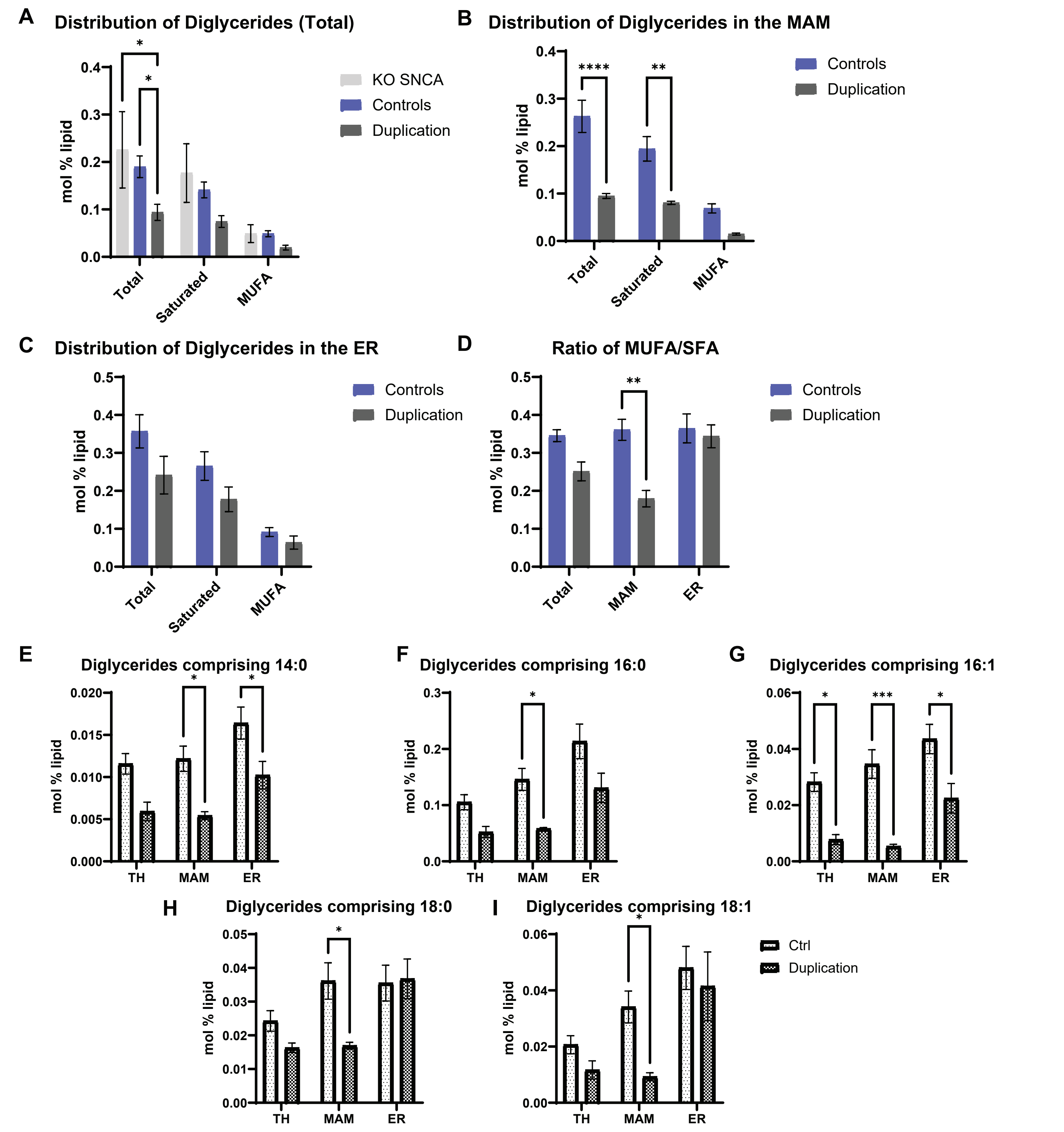

Supplementary Figure 6E: Alpha-synuclein level alters fatty acid preference for diglycerides across subcellular fractions. Distribution of diglycerides based on preference for saturated fatty acids (SFA) or monounsaturated (MUFA) in (**A**) total, (**B**) MAM and (**C**) ER subcellular fractions. The ratio of MUFA/SFA across subcellular fractions is shown in (**D**). Preference for diglycerides in different subcellular fractions containing specific fatty acids (**E**) (14:0 - Myristic Acid), (**F**) (16:0 - Palmitic Acid) (**G**) 16:1 - Palmitoleic Acid, (**H**) 18:0 - Stearic Acid and (**I**) 18:1 - Oleic Acid. For all statistical analyses, ordinary two-way ANOVAs were perform followed by either a Tukey's (**A-C**) or Sidak's (**E-I**) multiple comparison test with a single pooled variance. (**A**) Column (Cell line) factor ($F_{(2,36)}$=7.350; **p=0.0021), (**B**) Column (Cell line) factor ($F_{(2,36)}$= 24.79; ****p<0.0001), (**C**) Column (Cell line) factor ($F_{(2,36)}$= 4.072; *p=0.0255) (**D**) Column (Cell line) factor ($F_{(1,30)}$= 14.48; ***p=0.0006). (**E**) Column (Cell line) factor ($F_{(1,30)}$= 20; ***p=0.0001), (**F**) Column (Cell line) factor ($F_{(1,30)}$= 14.26; ***p=0.0007), (**G**) Column (Cell line) factor ($F_{(1,30)}$= 33.89; ****p<0.0001), (**H**) Column (Cell line) factor ($F_{(1,30)}$= 4.282; *p=0.0472), (**I**) Column (Cell line) factor ($F_{(1,30)}$= 5.720; *p=0.0232). Error bars reflect standard error of the mean. In all statistical analyses: *p<0.05, **p<0.01, ***p<0.001, ****p<0.0001.

Crude Mitochondrial Fraction

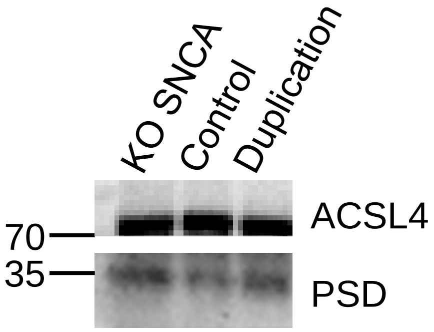

Supplementary Fig.7. Expression of PSD in iPS-derived neurons in a crude mitochondrial subcellular fraction
